# Legumain is a predictor of all-cause mortality and potential therapeutic target in acute myocardial infarction

**DOI:** 10.1038/s41419-020-03211-4

**Published:** 2020-11-26

**Authors:** Hui Yang, Yuhu He, Pu Zou, Yilei Hu, Xuping Li, Liang Tang, Zhaowei Zhu, Shi Tai, Tao Tu, Yichao Xiao, Mingxian Chen, Chenlu Wu, Shenghua Zhou

**Affiliations:** Department of Cardiology, the Second Xiangya Hospital, Central South University, Changsha, Hunan China

**Keywords:** Mechanisms of disease, Myocardial infarction

## Abstract

The prognostic impact of extracellular matrix (ECM) modulation and its regulatory mechanism post-acute myocardial infarction (AMI), require further clarification. Herein, we explore the predictive role of legumain—which showed the ability in ECM degradation—in an AMI patient cohort and investigate the underlying mechanisms. A total of 212 AMI patients and 323 healthy controls were enrolled in the study. Moreover, AMI was induced in mice by permanent ligation of the left anterior descending artery and fibroblasts were adopted for mechanism analysis. Based on the cut-off value for the receiver-operating characteristics curve, AMI patients were stratified into low (*n* = 168) and high (*n* = 44) plasma legumain concentration (PLG) groups. However, PLG was significantly higher in AMI patients than that in the healthy controls (median 5.9 μg/L [interquartile range: 4.2–9.3 μg/L] vs. median 4.4 μg/L [interquartile range: 3.2–6.1 μg/L], *P* < 0.001). All-cause mortality was significantly higher in the high PLG group compared to that in the low PLG group (median follow-up period, 39.2 months; 31.8% vs. 12.5%; *P* = 0.002). Multivariate Cox regression analysis showed that high PLG was associated with increased all-cause mortality after adjusting for clinical confounders (HR = 3.1, 95% confidence interval (CI) = 1.4–7.0, *P* = 0.005). In accordance with the clinical observations, legumain concentration was also increased in peripheral blood, and infarcted cardiac tissue from experimental AMI mice. Pharmacological blockade of legumain with RR-11a, improved cardiac function, decreased cardiac rupture rate, and attenuated left chamber dilation and wall thinning post-AMI. Hence, plasma legumain concentration is of prognostic value in AMI patients. Moreover, legumain aggravates cardiac remodelling through promoting ECM degradation which occurs, at least partially, via activation of the MMP-2 pathway.

## Introduction

Acute myocardial infarction (AMI) is a lethal event in cardiovascular disease (CVD)^1^. Cardiomyocyte necrosis occurs minutes after AMI, which can be detected based on increased serum troponin levels several hours after the attack. Macrophages and fibroblasts are the most abundant cells in the heart, which are involved in cardiac repair and remodelling^2^. However, the biochemical markers of cardiac remodelling and their mechanistic relationship with patient prognosis, remain elusive^3–5^.

Extracellular matrix (ECM) remodelling associated with the pathological processes following AMI. The cardiac ECM consists of collagens, glycoproteins, proteoglycans, among others^6^; of which, collagens, which are primarily produced by fibroblasts, are the most abundant^7^. Specifically, collagen type I and III are predominant elements responsible for the structural integrity of cardiomyocytes^8–10^; while disruption of the collagen network might result in cardiac rupture^11,12^. However, the mechanistic relevance of ECM remodelling in the context of AMI has not been fully characterised owing to the lack of mechanistic information regarding the molecular association between MI and ECM remodelling.

Legumain is a lysosome asparaginyl endopeptidase^13^ that is active in acidic conditions, and inactive in environments with PH > 6^14^. Moreover, legumain is significantly upregulated in tumour associated macrophages; this phenomenon is reportedly related to enhanced tumour metastasis through the activation of cathepsin, which possess collagenolytic and elastinolytic activities^15,16^. Legumain can also activate pro-MMP-2, thereby contributing to the degradation of ECM components, such as elastin, fibronectin, lamin, gelatine, and collagen^16–18^. In addition, legumain can directly degrade fibronectin in renal proximal tubular cells, resulting in ECM remodelling in the kidney^19^. Besides, legumain facilitates atherosclerotic plaque formation and plaque rupture via enhanced ECM degradation^20^. We, therefore, postulate that legumain could play a role in ECM degradation and might be an important mediator of cardiac injury and repair post-AMI. To date, the role of legumain in cardiac repair post-AMI and its prognostic value in AMI patients are not fully understood.

In this study, we evaluated the prognostic relevance of plasma legumain concentration (measured before coronary angiography) with respect to all-cause mortality in an AMI patient cohort and explored the mechanism by which legumain affects AMI pathogenesis by investigating legumain-mediated degradation of ECM in AMI mice and in vitro.

## Subjects and methods

### Patient population

A total of 212 consecutive patients, who were diagnosed as AMI and underwent coronary artery angiography between June 2015 and September 2016, were included in this study. AMI was diagnosed if a significant rise or fall in cardiac troponin T was observed, consistent with myocardial ischaemia^21^. Plasma legumain levels were assessed in these patients before coronary artery angiography and in 323 age- and sex-matched healthy controls. This study was performed according to the principles outlined in the Declaration of Helsinki. The research plan was approved by the ethics Committee of the Central South University. Informed consent was obtained from each participant at the beginning of the study.

### Study protocol

In all patients, peripheral venous blood was drawn at the time of hospital admission to measure serum creatinine (μmoL/L), N-terminal pro-brain natriuretic peptide (NT-proBNP, pg/mL), and high sensitivity cardiac troponin T (pg/mL). After overnight fasting, venous blood was collected to measure total cholesterol (mmoL/L), low density lipoprotein-cholesterol (LDL-C, mmoL/L), high density lipoprotein (HDL, mmoL/L), apoB (g/L), C reactive protein (CRP, mg/L), and glycosylated haemoglobin A1 (HbA1C, %). Cardiac troponin levels were tested repeatedly if necessary during hospitalisation. Before coronary angiography, blood was collected, and plasma was stored at −80 °C for legumain analysis.

Transthoracic echocardiography was performed during hospitalisation using a Vingmed Vivid 7 system (GE, Horten, Norway). Standard echocardiographic measurements for the evaluation of left atrial (LA) size and LV geometry and function were performed according to the American Society of Echocardiography (ASE) guidelines^22^. The left ventricular ejection fraction (LVEF, %) was calculated using Simpson’s biplane method.

Baseline demographic and clinical comorbidities were determined by reviewing the patients’ medical records. Patient death was confirmed by the official household registration survey, hospital files or from family members.

Plasma legumain (μg/L) was measured using a commercially available enzyme-linked immunosorbent assay (Quantikine; R&D System Inc, Minneapolis, MN, USA) according to the manufacturer’s instructions.

## Experimental studies

### Reagents

RR-11a, a synthetic specific legumain inhibitor, MG-132, and the commercial legumain substrate Z-Ala-Ala-Asn-7-amino-4-methylcoumarin (Z–Ala–Ala–Asn–AMC) were obtained from MedChemExpress. 2,3,5-Triphenyltetrazolium chloride (T8877), were purchased from Sigma-Aldrich (MO, USA). The MMP-2 inhibitor was obtained from Santa Cruz (SC-204092, 5 μmol/L; Santa Cruz Biotechnology, Santa Cruz, CA, USA). The legumain (sc-133234), collagen I antibody (sc-293182) and fibronectin (sc-8422) antibodies were purchased from Santa Cruz. The F4/80 antibody was purchased from Bio-Rad (Hercules, CA, USA). The α-SMA antibody (ab28052), collagen III antibody (ab7778), MMP-2 antibody (ab37150), and the mouse legumain ELISA kit (ab207617) were obtained from Abcam (Cambridge, UK). Mouse recombinant legumain (rLG) was obtained from Sino Biological, Inc. (Beijing, China), TGF-β1 was obtained from R&D Systems. Antibodies against HECTD1 (E3 ubiquitin-protein ligase, 20605-1-AP), GAPDH (60004-1-Ig), β-actin (60008-1-Ig), horseradish peroxidase HRP-conjugated Affinipure Goat Anti-Mouse IgG(H + L) (SA00001-1) and HRP-conjugated Affinipure Goat Anti-Rabbit IgG(H + L) (SA00001-2) were obtained from Proteintech Group (Rosemont, IL, USA).

### Mice

All animal experiments were conducted in accordance with the Guide for the Care and Use of Laboratory Animals by the US National Institutes of Health (NIH Publication No. 85–23, revised 1996) and approved by the Animal Care and use Committee of the Second Xiangya Hospital of Central South University. Eight to ten-week-old male C57BL/6J mice from SJA Laboratory Animal Co. Ltd (Changsha, China) were provided *ad libitum* access to water and sterile food under controlled temperature and light. The detailed study design is shown in Supplementary Figs. 1A and 1B. The sample size to be used was determined based on previous studies^23,24^. MI was induced by left coronary ligation in 215 mice, and sham operation was performed in 56 mice without left coronary ligation. Echocardiography was performed for all mice, by an operator, blinded to the procedure, 24 h after operation. MI mice with ejection fraction ≤ 55% were selected for further experiments. To further identify the homogeneity of the MI model, left coronary artery ligation was performed in ten mice that were then randomly divided into two groups using a random number table. Twenty-four hours after ligation, mice were euthanised by narcotic overdose and TTC staining was performed to measure the infarct size in all mice. MI group mice (*n* = 215) received daily intraperitoneal injections of RR-11a (*n* = 84) at a dose of 20 mg/kg body weight, or an equal volume of saline (*n* = 131) on the second day after MI, and daily thereafter, for 29 days. Meanwhile, 22 mice in the sham group were injected with RR-11a after operation and the others were injected equal volume of saline (*n* = 34). The legumain inhibitor, RR-11a^25,26^ (MedChemExpress, Shanghai, China) was freshly prepared by dissolving in normal saline before administering to animals. Blood was obtained at 1, 3, 7, and 14 days post-operation as described previously^27,28^ to measure plasma legumain levels. To test the pharmacokinetics of RR-11a—0.25, 0.5, 1, 2, 4, 6, 8, 12 and 24 h after intraperitoneal injection—its plasma concentration was measured in MI mice 3 days post-operation in triplicate. To investigate whether the activity or expression of legumain was inhibited by daily injection of RR-11a, its activity, plasma concentration, and infarct tissue expression were measured 20 h after RR-11a injection 3 and 14 days post-MI in triplicate. Legumain expression and activity were also assessed in vitro.

### Myocardial infarction model

Permanent ligations of the left anterior descending artery (MI) or sham operations without ligation were performed as described previously^29^. Briefly, mice were anaesthetised with 2% isoflurane inhalation with an isoflurane delivery system (Viking Medical, Medford, NJ). The left coronary artery was sutured about 3 mm distant from the LA appendage with a 6–0 silk. Sham-operated animals underwent the same procedure without coronary artery ligation.

### Mice echocardiography

Transthoracic echocardiography of animals was performed with a Vinno 6 lab instrument (VINNO Technology (Suzhou) Co.,Ltd.) with an X10-23L transducer as previously described^30^. Mice were anaesthetised with 2% isoflurane inhalation. M-mode tracings were recorded through the anterior and posterior LV walls at the papillary muscle level to measure the LVEDD, LV end-systolic dimension (LVESD), LV fractional shortening (FS), End-diastolic volume (EDV), end-systolic volume (ESV), and ejection fraction (EF).

### 2,3,5-Triphenyltetrazolium chloride (TTC) staining

Twenty-four hours after MI, mice were euthanized and the heart was then placed in a −20 °C freezer for 10 min. Short-axis cuts of the heart at 1 mm thickness were incubated at 37 °C in 1% TTC for 30 min. Heart sections were fixed in 2% Paraformaldehyde, each stained cardiac section was photographed and weighed. With this procedure, the infarcted tissue was white. The infarcted area was analyzed using ImageJ software (NIH, Bethesda, MD), and from these measurements the infarct size was calculated as a percentage of the infarcted region to LV using a weight-based method^29^.

### Infarct size and infarct wall thickness determination

After 30 days post operation, mice were sacrificed and heart tissue samples were fixed in formalin for 24 h, dehydrated through a graded ethanol series, embedded in paraffin, and cut into 5 μm thick sections. Picrosirius red and Masson’s trichrome staining of the paraffin-embedded sections was then performed according to the manufacturer’s protocol (Servicebio, Wuhan, China). Sections from the mid-level were used to determine the morphology and infarct size, with the latter calculated as the total infarct area divided by the total LV wall (including both the free wall and septal portion) area × 100%. The free wall thickness of the scars at the papillary and apical levels was measured according to a previously described method^31^. The fraction of collagen volume was assessed in ten randomly chosen high-power fields (200×) in each section. These data were analysed using Image J software.

### Immunofluorescence staining

For immunofluorescence staining, frozen cardiac tissues from the infarct area were cut into 5 μm thick sections and mounted on adhesion slides. Sections were fixed with cold methanol for 20 min, blocked in 10% goat serum at room temperature for 1 h to prevent nonspecific binding and then incubated with antibodies against legumain (sc-133234), F4/80, α-SMA (ab28052), collagen I (ab34710), collagen III (ab7778), and MMP-2 (ab37150) overnight at 4 °C, followed by incubation with the Alexa Fluor-594 or 488 conjugated secondary antibodies, as appropriate, (1:1000; Invitrogen, Carlsbad, CA, USA) for 1 h at room temperature. Nuclei were stained with DAPI. The sections were sealed with an antifade reagent and observed under an Olympus BX61 microscope (Tokyo, Japan) or a Zeiss LSM780 laser scanning confocal microscope (Carl Zeiss, Oberkochen, Germany).

### Culture and stimulation of murine cardiac fibroblasts

Cardiac fibroblasts (CFs) were isolated from the heart of 8- to 10-week-old males C57BL/6J mice^23^. Cardiac fibroblasts at passage 3–6 were used for TGF-β1(10 ng/ml) stimulation experiments. To assess the ECM degradative capability of legumain, we added rLG to the medium of TGF-β1-treated CFs. To demonstrate the specificity of legumain, the legumain inhibitor RR-11a (100 nM) was added to the rLG. The level of fibrotic proteins in cardiac fibroblast lysates or culture media were then detected by western blotting.

### Quantitative real-time PCR

Total RNA samples from cardiac tissues of infarct area and infarct border zone were prepared using Trizol reagent (Invitrogen, Carlsbad, CA, USA), according to the manufacturers’ instructions. A first-strand cDNA synthesis kit (Invitrogen) was used for cDNA synthesis. Real-time qPCR was performed using the SYBR Green mix (Applied Biosystems, Foster City, CA, USA). The relative expression of target genes was normalised to that of the housekeeping gene GAPDH.

### Western blotting

Heart tissue samples from infarct area or murine CFs were homogenised in RIPA lysis buffer (CW2333, CWBIO, Jiangsu, China) containing a proteinase and phosphatase inhibitor cocktail. Equal amounts of proteins were loaded onto SDS-PAGE gels and after electrophoresis were electro-transferred onto PVDF membranes (Millipore). The membranes were incubated with primary antibodies overnight at 4 °C before blocking with 5% BSA (for phosphorylation-specific antibodies) or 5% skimmed milk (for the other antibodies) at room temperature for 2 h, followed by horseradish peroxidase (HRP)-conjugated secondary antibodies (1:5000, Proteintech Group, Rosemont, IL, USA). Antibody labelling was visualised using an electrochemiluminescence (ECL) system (GE Healthcare Biosciences, Pittsburgh, PA, USA). GAPDH or β-actin served as loading controls. The densitometric analysis of positive bands was performed using Image J software.

### Legumain activity assay

The activity of legumain in cardiac tissue, plasma and supernatant of cardiac fibroblast were detected. Legumain activity assay was performed as previously described^25^. The commercial legumain substrate Z–Ala–Ala–Asn–AMC was used to evaluate the activity of legumain by comparing the AMC signals in the samples. Ten microlitre samples of plasma, cardiac tissue homogenates or supernatant of cardiac fibroblast were added to 96-well plate and incubated with 190 μl Z–Ala–Ala–Asn–AMC (the final concentration is 10 μM) at 37 °C for 2 h. Then, the activity of legumain was examined by measuring the fluorometrical number (excitation, 360 nm; emission, 440 nm) of liberation of AMC.

### Gelatin zymography

Conditioned culture media from CFs were collected and prepared in a standard, non-reducing 2× loading buffer for SDS-PAGE. Gelatin liquor (1%, 0.5 mL) was embedded in the separation gel (10%, 5 mL) during preparation of the acrylamide gel. Following electrophoresis, the SDS was removed from the gel by washing three times for 25 min with a buffered 2.5% Triton X-100 solution, followed by incubation in an appropriate digestion buffer (pH 7.6, 50 mM Tris-HCl, 10 mM CaCl2, and 5 mM NaCl) for 42 h at 37 °C. The gel was subsequently stained with Coomassie Brilliant Blue, and areas of digestion appeared as white bands against a blue background, the areas of which were proportionate to the MMP-2 activity.

## Statistical analysis

Statistical analysis was performed using IBM SPSS for Windows (version 25), the statistical software package R (http://www.R-project.org, The R Foundation), EmpowerStats (http://www.empowerstats.com, X&Y Solutions, Inc., Boston, MA, USA), and GraphPad Prism 5.0 (Graph Pad Prism Software Inc., San Diego, CA, USA). A two-tailed probability value < 0.05 was considered significant. Continuous data are expressed as mean ± standard deviation (SD) or standard error of the mean (SEM)—for normal distribution—or median and interquartile range —for skewed distribution—and categorical variables as count (percent). The normal distribution of continuous variables was examined by inspecting skewness, kurtosis, and Q–Q plots. The cut-off value of plasma legumain levels for all-cause mortality derived from the receiver-operating characteristic (ROC) curve was 10.1 μg/L (Supplementary Fig. 2). Patients were assigned to low or high plasma legumain groups based on this cut-off value. Differences in continuous data between two groups were compared using a Student´s *t* test—for normal distribution data after homogeneity of variance test—or by a Mann–Whitney rank sum test for skewed distribution data. Categorical variables were compared using Chi-square and Fisher’s exact tests, as appropriate.

Unadjusted model was first employed for analysing the influence of plasma legumain on patient prognosis, then age and sex were included in the minimally adjusted model (model I). To evaluate the independent risk of legumain on AMI patients, clinical variables except NT-proBNP, when added to the minimally adjusted model, changed the matched hazard ratio by at least 10%, were included as confounding variables in the fully adjusted model (model II). The confounders in model II are as follows: age, sex, body mass index, left ventricular end diastolic diameter (LVEDD), low density lipoprotein, peak cardiac troponin T level, LVEF and multi-vessel disease.

Kaplan–Meier curves were generated according to the low and high plasma legumain levels. Correlations between plasma legumain with NT-proBNP, CRP, LVEDD and LA diameter were assessed by Pearson’s test.

The relative prognostic value of CRP, NT-proBNP, LVEDD, or LVEF vs. PLG was assessed by comparing the areas under ROCs curves (AUC). An ROC curve represents sensitivity vs. 1-specificity (or false-positive rate) for various cut-off definitions of a positive diagnostic test result^32^. Statistical differences in the AUCs were compared using the method of DeLong et al.^33^.

## Results

### Clinical study

#### Baseline characteristics of the participants

A total of 131 STEMI and 81 NSTEMI patients comprised this cohort. Thirty-five (15.1%) patients died during a median follow-up of 39.2 months (interquartile range: 34.0–42.8 months).

Age and sex did not differ between AMI patients and healthy controls. The plasma levels of legumain (PLG) were significantly higher in the AMI group compared to those in the healthy group (median 5.9 μg/L, interquartile range: 4.2–9.3 μg/L in AMI group vs. median 4.4 μg/L, interquartile range: 3.2–6.1 μg/L in control group, *P* < 0.001; Supplementary Table 1).

**Fig. 1:**
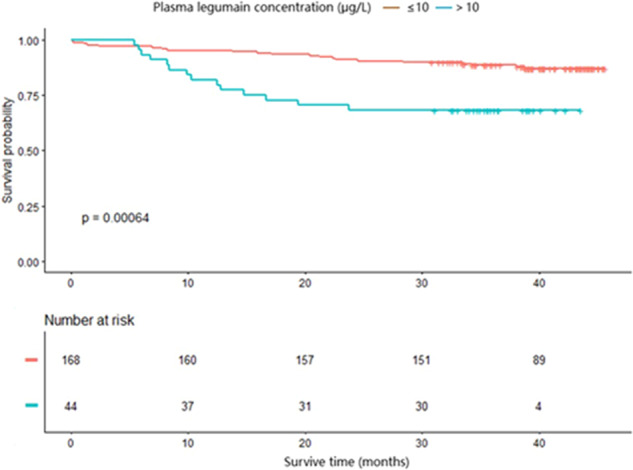
Survival analysis between low and high plasma legumain group. Kaplan–Meier estimates of all-cause mortality between AMI patients with low (≤10μg/L) and high (>10μg/L) plasma legumain levels.

AMI patients were further divided into low (*n* = 168) and high legumain groups (*n* = 44) based on the ROC curve cut-off value. Age, sex, body mass index, and most clinical characteristics were comparable between the two groups, while heart failure and diabetes mellitus incidence, as well as glycosylated haemoglobin A1C and N-terminal pro-brain natriuretic peptide (NT-proBNP) levels were significantly increased in the high legumain group compared to those in the low legumain group (all *P* < 0.05; Table 1). Despite similar hospital mortality rates, the all-cause mortality was significantly higher in high legumain group than that in the low legumain group (Fig. 1).

**Table 1:** Baseline characteristic of participants in patients with myocardial infarction with heart failure.

Characteristic	Low PLG (≤10 μg/L)	High PLG (>10 μg/L)	*P* value
N	168	44	
Demographic data			
Age (years)	60.4 ± 12.9	63.0 ± 11.5	0.228
Male sex, n(%)	133 (79.2%)	33 (75.0%)	0.551
Body mass index	24.1 ± 3.5	24.0 ± 3.4	0.949
Smoking, n(%)	92 (56.8%)	27 (62.8%)	0.478
Past medical history, n (%)			
Diabetes mellitus	35 (20.8%)	15 (34.1%)	0.053
Hypertension	93 (55.4%)	26 (59.1%)	0.657
History of heart failure	46 (27.4%)	21 (47.7%)	0.01
Physical and laboratory test			
Heart rate (bpm)	78.1 ± 14.2	82.5 ± 13.4	0.063
Plasma legumain concentration (μg/L)	4.9 (3.9–7.1)	12.0 (10.9–13.6)	<0.001
HbA1C (%)	6.2 (5.6–7.6)	7.8 (6.3–9.1)	0.006
Serum creatinine (μmol/L)	82.0 (68.7–98.9)	87.6 (65.7–114.9)	0.455
Ccr (mL/min)	76.9 (53.5–99.9)	68.0 (43.4–98.9)	0.291
High sensitive CRP (mg/L)	7.5 (3.4–27.7)	13.0 (5.1–42.3)	0.062
Cardiac troponin T (pg/mL)	725.9 (126.8–2389.8)	734.1 (112.2–2937.2)	0.886
NT-proBNP (pg/mL)	772.5 (379.3–1590.5)	2419.0 (1081.2–6250.3)	<0.001
Total Cholesterol (mmoL/L)	4.1 ± 0.9	4.3 ± 1.0	0.202
Low density lipoprotein (mmoL/L)	2.5 ± 0.9	2.8 ± 0.8	0.113
High density liporotein (mmoL/L)	1.0 ± 0.3	1.0 ± 0.3	0.996
ApoB (g/l)	0.9 ± 0.3	1.0 ± 0.3	0.125
Echocardiography test and coronary angiography			
Left ventricular end diastolic diameter (mm)	49.6 ± 5.3	52.4 ± 6.0	0.003
LA diameter (mm)	36.2 ± 6.1	38.9 ± 4.8	0.007
Left ventricular ejection fraction (%)	50.3 ± 8.6	48.6 ± 10.4	0.303
Tricuspid valve regurgitation velocity (m/s)	2.3 (2.0–2.6)	2.4 (2.0–2.7)	0.292
Multi-vessel disease	134 (80.7%)	40 (90.9%)	0.122
Medication and clinical outcomes, n (%)			
Aspirin	156 (92.9%)	43 (97.7%)	0.311
Beta blocker	127 (75.6%)	35 (79.5%)	0.583
Statins	157 (93.5%)	43 (97.7%)	0.467
ACEI or ARB	124 (73.8%)	33 (75.0%)	0.873
CABG	2 (1.2%)	1 (2.3%)	0.504
Stent implantation	113 (67.3%)	32 (72.7%)	0.488
Hospital death	4 (2.4%)	1 (2.3%)	0.966
All cause death	21 (12.5%)	14 (31.8%)	0.002

Data are shown as: Mean ± SD or Median (Q1–Q3) or N (%).

*PLG* plasma legumain concentration, μg/L, *HbA1C* glycosylated haemoglobin A1C, *CRP* C reactive protein, *NT-proBNP* N terminal pro-brain natriuretic peptide, *LA* left atria, *ACEI* angiotensin converting enzyme inhibitor, *ARB* angiotensin receptor blocker, *CABG* coronary artery bypass grafting, *Ccr* endogenous creatinine clearance rate.

#### Multivariate Cox regression analysis

Multivariate Cox regression was used to investigate the independent predictive effect of legumain on prognosis. In the crude model, the hazard ratio for the effect of high plasma legumain levels on all-cause mortality was 3.1, with a 95% CI from 1.6 to 6.1 (*P* = 0.001; Table 2). After adjusting for age and sex, high plasma legumain remained an independent risk factor of all-cause mortality (HR = 2.9, 95% CI: 1.5–5.8, *P* = 0.002). In model II, after adjusting for age, sex, body mass index, LVEDD (mm), multi-vessel disease, LVEF (%), low density lipoprotein (mmoL/L), and peak cardiac troponin T level (pg/mL), the HR of the effect of plasma legumain concentration on all-cause death was 3.1, the 95% CI was 1.4–7.0 and *P* value was 0.005.

**Table 2:** Multivariable Cox regression of plasma legumain concentration on all cause death.

Exposure	Non-adjusted	Adjust I	Adjust II
PLG (μg/L)	1.1 (1.0, 1.2) 0.011	1.1 (1.0, 1.2) 0.010	1.2 (1.0, 1.3) 0.005
PLG subgroup			
≤10 μg/L	1.0	1.0	1.0
>10 μg/L	3.1 (1.6, 6.1) 0.001	2.9 (1.5, 5.8) 0.002	3.1 (1.4, 7.0) 0.005

Data are expressed as: HR (95% CI) *P* value. Non-adjusted model adjust for: None. Adjust I model adjust for: age (years), sex. Adjust II model adjust for: age (years), sex, body mass index, left ventricular end diastolic diameter (mm), multi-vessel disease, left ventricular ejection fraction (%), low density lipoprotein (mmoL/L), Peak cardiac troponin T level (pg/mL).

#### Correlation analyses

Pearson’s correlation analysis determined that plasma legumain levels correlated positively with NT-proBNP ln transform (*r* = 0.324; *P* < 0.0001), LA diameter (*r* = 0.2232; *P* = 0.0015), and LVEDD values (*r* = 0.1855; *P* = 0.0087), but not with CRP levels (Pearson’s *r* = 0.1155; *P* = 0.1447; Supplementary Fig. 3).

#### Comparison of AUC under ROC curve between CRP, NT-proBNP, LVEDD, or LVEF vs. PLG

Comparison of the overall prognostic significance of PLG vs. CRP, NT-proBNP ln transform, LVEDD, and LVEF—as assessed by the area under the ROCs curve—showed that PLG (area: 0.66395, Harrell’s C index 0.598, 95% CI [0.4896–0.7065]) exhibited similar prognostic power as LVEDD (area: 0.69326, Harrell’s C index 0.6178, 95% CI [0.51–0.7256]). Compared to LVEF (area: 0.60334, Harrell’s C index 0.6387, 95% CI [0.5243–0.753]), PLG exhibited similar prognostic value (area: 0.66288, Harrell’s C index 0.5965, 95% CI [0.4878–0.7051]). However, ln NT-proBNP (area: 0.75816, Harrell’s C index 0.7861, 95% CI [0.7165–0.8558]) exhibited better prognostic efficiency than PLG (area: 0.65293, Harrell’s C index 0.6059, 95% CI [0.5061–0.7057]). The AUC tended to be higher for PLG (area: 0.71489, Harrell’s C index 0.6166, 95% CI [0.5041–0.7291]) than that for CRP (area: 0.5109, Harrell’s C index 0.618, 95% CI [0.5006–0.7353]; however, the overlap between the 95% CI of the two AUCs indicates insignificance (Supplementary Table 2).

### In vitro and in vivo study

#### Legumain is upregulated in cardiac tissue in response to myocardial infarction

**Fig. 2:**
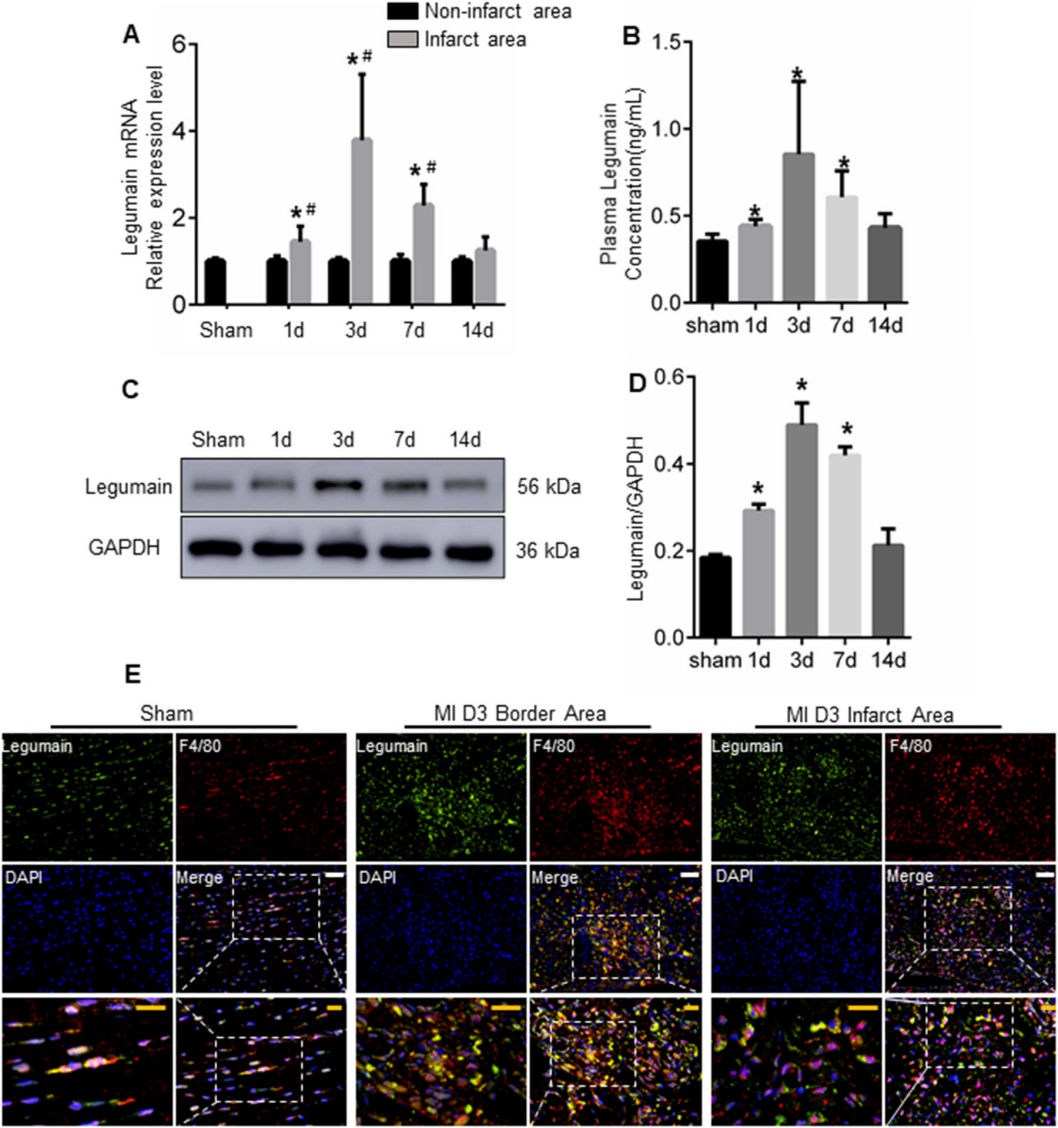
Legumain is upregulated in the heart tissue following MI induction in a mouse model. **A** Quantitative analysis of legumain mRNA levels in infarct and non-infarct areas at different time points after MI induction; *P < 0.05 vs. sham; ^#^*P* < 0.05 vs. Non-infarct area; (Sham: *n* = 6; 1d: *n* = 7; 3d: *n* = 6; 7d: *n* = 8; 14d: *n* = 4). **B** Changes in plasma legumain levels over time after MI; *P < 0.05 vs. sham; *n* = 8. **C**–**D** Legumain protein levels were determined in cardiac tissues post-MI at different time points. *P < 0.05 vs. sham; *n* = 4. **E** Representative immunofluorescence images of legumain (green) and F4/80 staining in mouse hearts on day 3 post-MI, *n* = 3. Scale bars: white, 50 μm; yellow, 20 μm.

To determine if legumain is involved in cardiac remodelling post-MI, we assessed peripheral legumain concentration and post-MI cardiac tissue expression in mice. Consistent with the clinical data, both peripheral concentration and cardiac expression of legumain were low in healthy heart and increased gradually post-MI, peaking on day 3 post-MI (Fig. 2A–D). Moreover, immunofluorescence identified the colocalisation of legumain (green) and macrophage marker F4/80 (red) in the infarct and in the infarct border zone (Fig. 2E). Hence, increased peripheral and cardiac legumain expression is associated with increased macrophage infiltration post-MI.

#### Legumain activity, not expression, is inhibited in response to RR-11a treatment

**Fig. 3:**
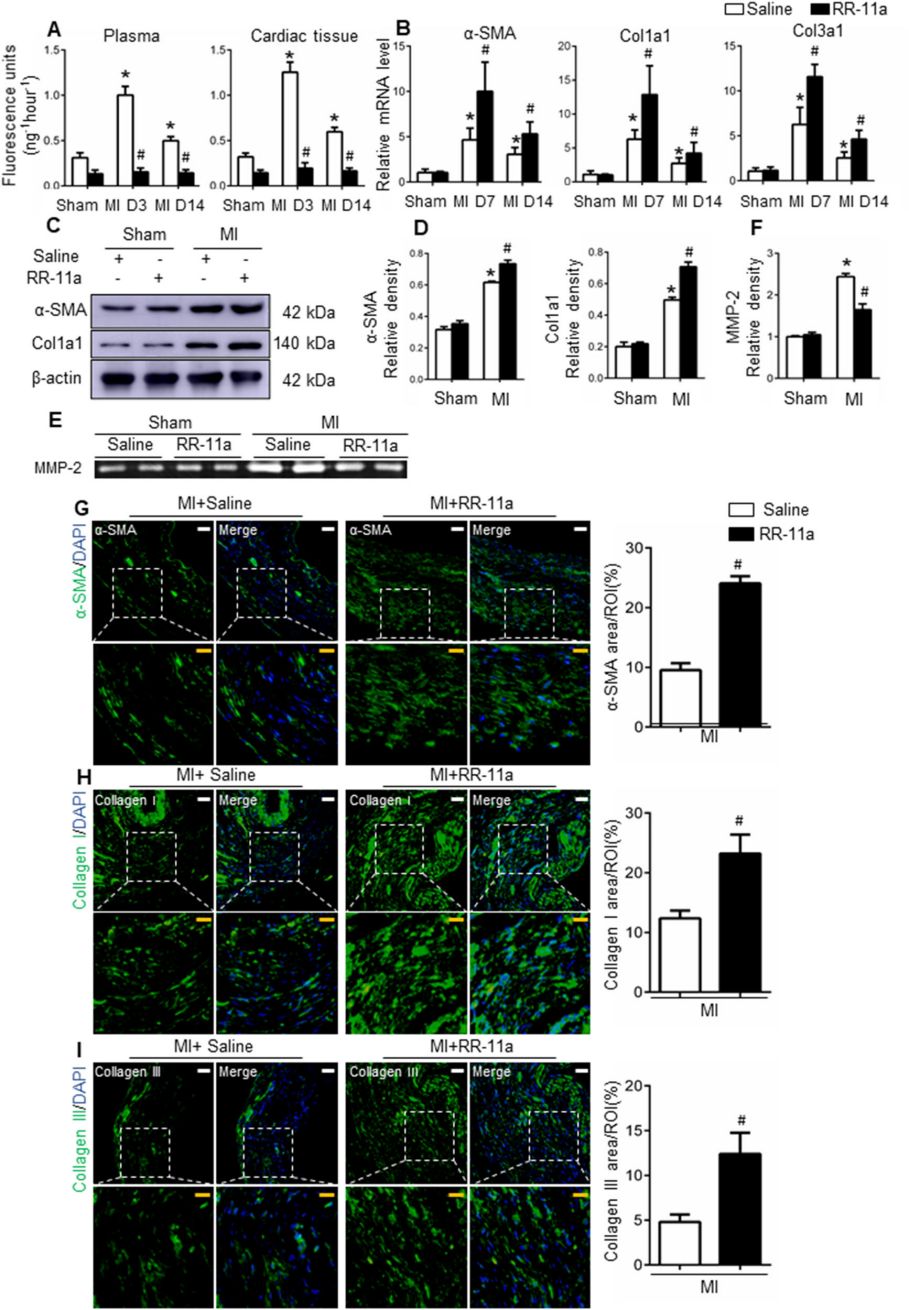
Effects of RR-11a on wound healing and scar formation after MI. **A** Legumain activity in plasma and cardiac tissue collected from saline-treated or RR-11a-treated mice at 3 and 14 days, *n* = 4. **B** Relative mRNA levels of α-SMA, collagen I and III in the cardiac tissue collected from saline-treated or RR-11a-treated mice at 7 and 14 days, *n* = 4. **C**–**D** Protein levels of α-SMA and collagen I in hearts from saline-treated and RR-11a-treated mice, with or without MI on day 7, *n* = 4. **E**–**F** Gelatine zymogram analysis of MMP-2 activity in cardiac tissues from saline-treated and RR-11a-treated mice on day 3 after sham operation or after induction of MI, *n* = 3. **G**–**I** Representative immunofluorescence images of α-SMA (**G**), collagen I (**H**), and collagen III (**I**) in cardiac tissues from saline-treated or RR-11a-treated mice on day 30 post-MI, *n* = 4. Scale bars: white, 50 μm; yellow, 20 μm. *P < 0.05 vs. sham mice; ^#^*P* < 0.05 vs. saline-treated MI mice. Col1a1: collagens I; Col3a1: collagens III.

**Fig. 4:**
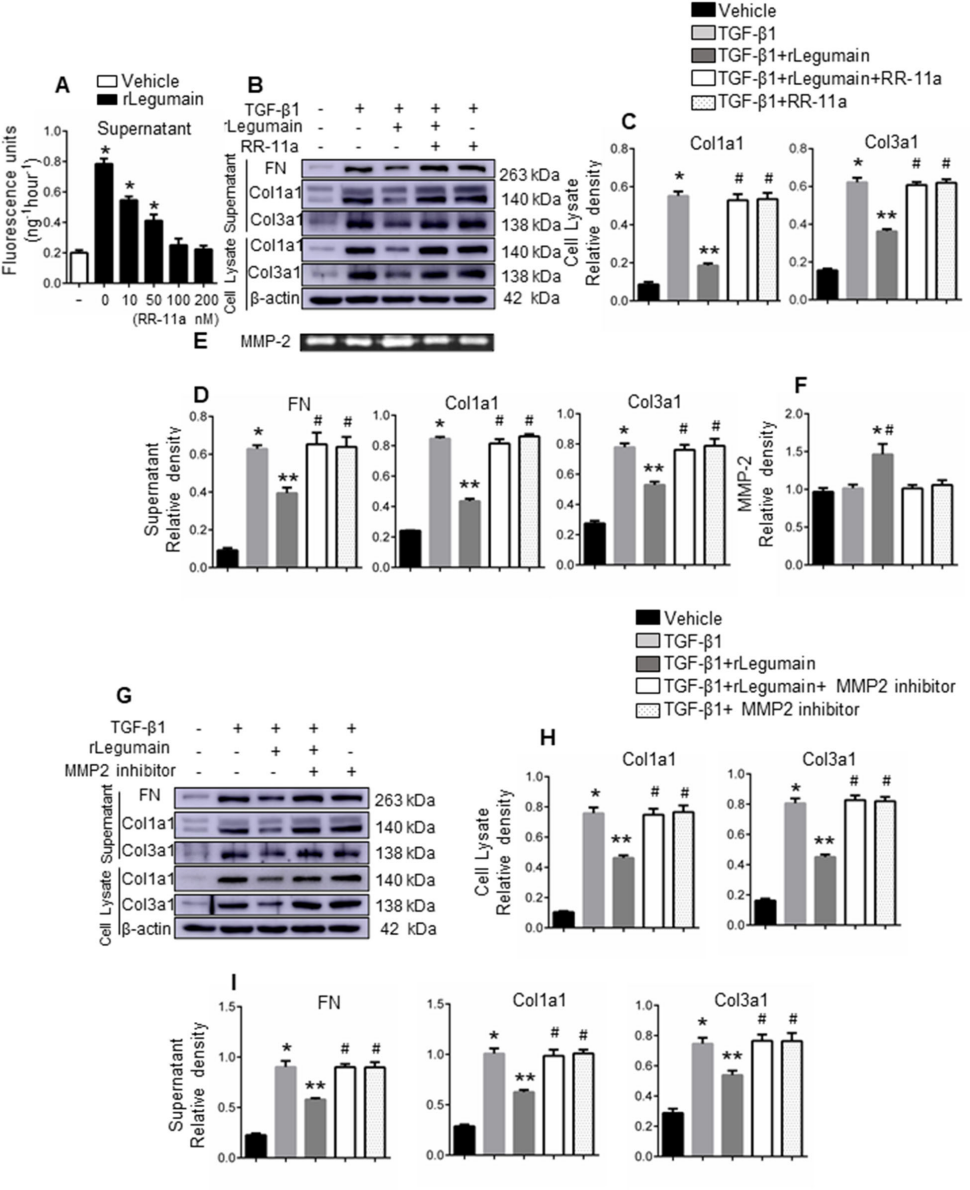
Legumain induces extracellular matrix degradation by activating MMP-2. **A** Activity of legumain in culture media after treatment with or without recombinant legumain and different concentrations of RR-11a, *n* = 3. **B** Western blot analysis of fibronectin, collagen I, and collagen III expression in TGF-β1-treated cardiac fibroblasts lysates and culture media after treatment with recombinant legumain, with or without RR-11a. **C**–**D** Quantification of collagen I, and collagen III in cell lysates (**C**) and supernatant (**D**) in (**A**), *n* = 4. **E**–**F** Gelatine zymogram for investigating MMP-2 activity in the supernatant of TGF-β1-treated cardiac fibroblasts after treatment with recombinant legumain, with or without RR-11a, *n* = 3. **G** Western blot analysis of fibronectin, collagen I, and collagen III expression in TGF-β1-treated cardiac fibroblast lysates and supernatant after treatment with recombinant legumain, with or without an MMP-2 inhibitor. **H**–**I** Quantification of fibronectin, collagen I, and collagen III in supernatant and cell lysates in (**G**), *n* = 4. *P < 0.05 vs. salinee treatment, **P < 0.05 vs. TGF-β1 treatment, ^#^*P* < 0.05 vs. TGF-β1 and recombinant legumain treatment. rLegumain: recombinant legumain, Col1a1: collagens I, Col3a1: collagens III.

To investigate whether the expression or activity of legumain was inhibited by RR-11a, we monitored its concentration after intraperitoneal RR-11a injection (Supplementary Fig. 4). The concentration of RR-11a peaked 1.5 h after injection and the plasma half-life was ~5 h. We then detected legumain expression and concentration in myocardium and plasma 20 h after RR-11a injection in MI mice 3, and 14 days post-operation. Legumain expression and plasma concentration were similar between RR-11a and saline treatment groups (*P* > 0.05; Supplementary Fig. 5). Notably, the activity of legumain was significantly decreased after RR-11a treatment in both cardiac tissue and peripheral circulation (Fig. 3A). Legumain was also weakly expressed in cardiac fibroblast cells (Supplementary Fig. 6A); however, it’s expression in fibroblasts did not differ in the absence or presence of RR-11a (Supplementary Fig. 6B). The activity of legumain was dose-dependently inhibited by RR-11a, and 100 nM RR-11a sufficiently inhibited the legumain activity (Fig. 4A).

#### Legumain inhibition increases the fibroblast and collagen content in the infarcted myocardium

The expression levels of fibroblast marker α-smooth muscle actin (α-SMA) increased gradually and peaked on day 7 in the infarct area; this was in agreement with the increased expression of collagen I and collagen III (Fig. 3B) and demonstrated the self-repairing process inside the myocardium post-MI. Meanwhile, RR-11a further increased the expression of α-SMA, collagen I and collagen III (Fig. 3B–D). Immunofluorescence showed that α-SMA, collagen types I and III were all increased following RR-11a treatment (Fig. 3G–I). Taken together, these results indicate that legumain activation promotes collagen degradation in vivo.

#### Legumain facilitates TGF-β induced collagen and fibronectin degradation in fibroblasts in vitro

TGF-β is a key cytokine related to the response of the heart post-MI^34^; it induces the proliferation of CFs, phenotypic transformation of CFs into myofibroblasts, and ECM deposition^35^. Fibronectin is another main component of the ECM that may be degraded by legumain^19^. Here, TGF-β was used as a stimulator of fibronectin and collagen synthesis in CFs to assess the contribution of legumain to ECM degradation. As expected, reconstructed legumain significantly decreased the expression of fibronectin and collagen in the culture media and cell lysates. Furthermore, these effects were partially reversed by treatment with RR-11a (Fig. 4B–D). Hence, legumain facilitates collagen and fibronectin degradation in cardiac fibroblast cells.

#### Legumain facilitates collagen and fibronectin degradation through MMP-2

MMP-2 is the most abundant matrix metalloproteinase in the cardiac tissue and is known to mediate pathological remodelling post-MI. Accordingly, MMP-2 activity was significantly increased in cardiac tissues post-MI in mice, and RR-11a decreased this activity (Fig. 3E–F). Recombinant legumain increased MMP-2 activity and RR-11a decreased MMP-2 activity in TGF-β-treated fibroblasts (Fig. 4E–F). Furthermore, the MMP-2 positive area in the infarcted zone as well as in the infarcted border zone was increased at all observed points post-MI (Supplementary Fig. 7). In contrast to legumain, MMP-2 inhibitor significantly increased fibronectin and collagen I/III content—in cell supernatants—and collagen I/III expression in the lysates of fibroblast cells (Fig. 4G–I). Thus, MMP-2 might play a vital role in legumain-mediated fibronectin and collagen I/III degradation.

#### Legumain mediates collagen and fibronectin degradation and E3 ubiquitin ligases

HECTD1 (E3 ubiquitin-protein ligase), which encodes a novel protein homologous to the E6-AP C-terminal (HECT) domain-containing E3 ubiquitin (Ub) ligase, is expressed in human blood cells, liver, and heart and plays an important role in the ubiquitin-proteasome system (UPS)^36^. HECTD1 participates in the regulation of pulmonary fibrosis through ubiquitination^37^. Here, we investigated the association between HECTD1 and legumain-associated ECM degradation in an MI mouse model. Post-MI, HECTD1 was upregulated in the cardiac tissue; however, no significant difference was observed between saline and RR-11a-treated groups (Supplementary Fig. 8A–B). Western blotting revealed that legumain-mediated degradation of COL1A1 and COL3A1—in fibroblasts—and fibronectin, COL1A1, COL3A1 in culture supernatants was similar in the absence or presence of the E3 inhibitor (Supplementary Fig. 8C–E). Hence, the ubiquitination pathway does not play a fundamental role in legumain-mediated ECM degradation post-MI.

#### Legumain inhibition ameliorates MI-induced cardiac remodelling and reduces the cardiac rupture rate by increasing the ECM content

**Fig. 5:**
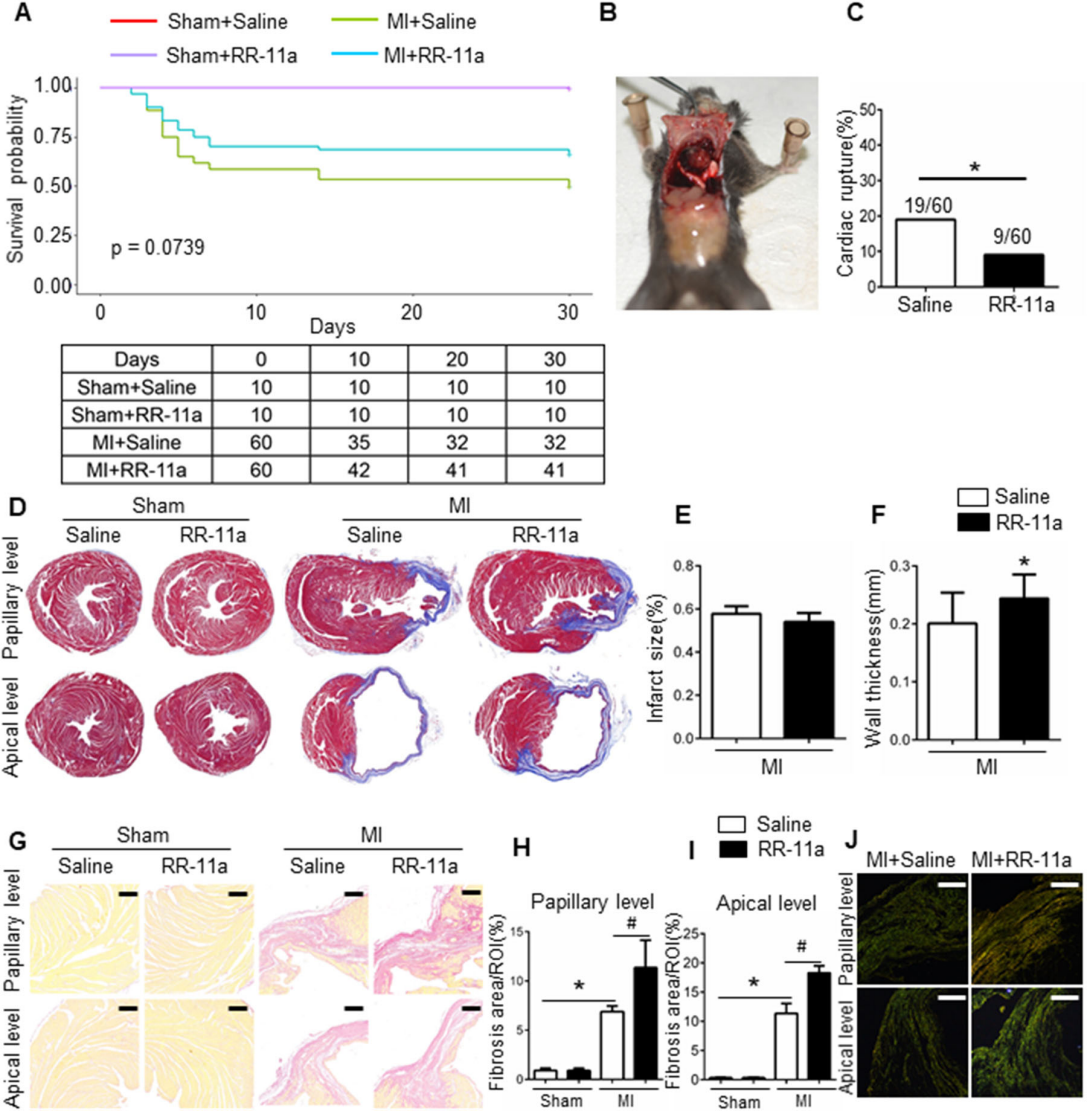
Effects of RR-11a on survival rate, wall thickness, and myocardial fibrosis. **A** Kaplan–Meier survival analysis at different time points after MI. **B** Representative ventricular rupture 3 days after the induction of MI. **C** Incidence of cardiac rupture in saline-treated and RR-11a-treated mice on day 30 post-MI. **D** Representative Masson’s trichrome staining of hearts collected from saline-treated and RR-11a-treated mice on day 30 post-MI or sham operation. **E**–**F** Quantitative analysis of infarct size (**E**), and wall thickness (**F**) using Masson’s trichrome staining. *P < 0.05 vs. saline-treated MI mice, *n* = 8. **G** Representative picrosirius-red–stained cardiac tissue from saline-treated and RR-11a-treated mice on day 30 post-MI or sham operation; the red staining represents collagens I and III. **H**–**I** Quantification of fibrotic areas at papillary (**H**) and apical (**I**) levels 30 days post-MI in saline-treated and RR-11a-treated mice; *P < 0.05 vs. sham-operated mice; ^#^*P* < 0.05 vs. saline-treated MI mice; *n* = 8. **J** Representative polarised microscope images of picrosirius-red–staining, collagen bundles appear green or yellow (*n* = 8). Scale bars: white, 50 μm; black, 50 μm.

**Fig. 6:**
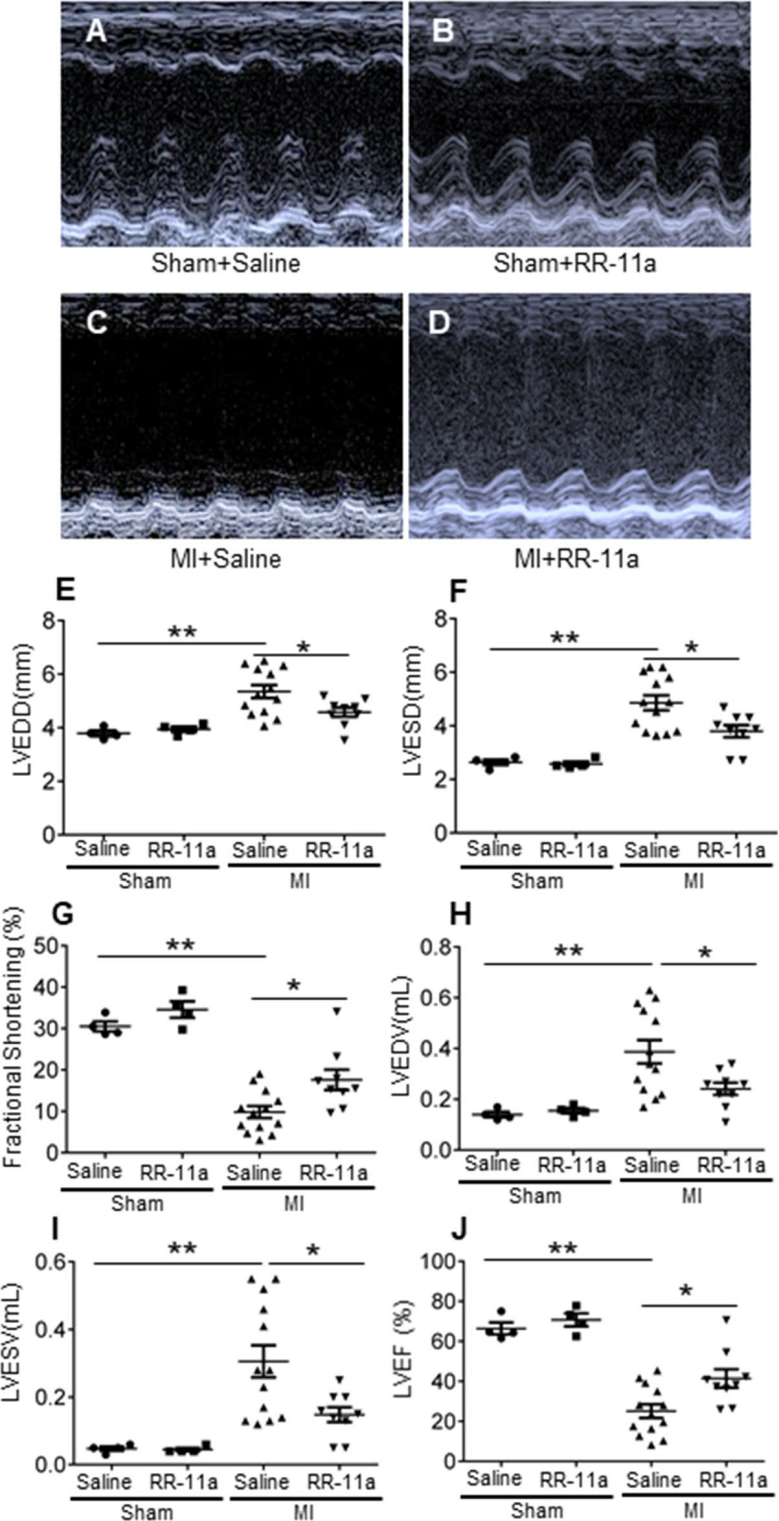
Comparison of cardiac function between saline-treated and RR-11a-treated mice 30 days post-MI. **A**–**D** Representative images representing echocardiographic analysis of left ventricle. **E** Echocardiographic analysis of left ventricular end diastolic diameter (LVEDD). **F** Echocardiographic analysis of left ventricular end systolic diameter (LVESD). **G** Echocardiographic analysis of fractional shortening (FS). **H** Echocardiographic analysis of left ventricular end diastolic volume (LVEDV). I Echocardiographic analysis of left ventricular end systolic volume (LVESV). **J** Echocardiographic analysis of left ventricular ejection fraction (LVEF). *P < 0.05; **P < 0.01.

To evaluate the homogeneity of left coronary ligation-induced MI model, we performed TTC staining in ten mice, randomly divided into two subgroups 24 h after operation. The infarcted volume/total LV volume was 35.79 ± 0.87% in control group and 36.14 ± 1.1% in the RR-11a-treatment group (*P* = 0.8139; Supplementary Fig. 9), suggesting that our mouse MI model was stable. In addition, only mice with EF ≤ 55% on day 1 post-MI were included in the study and were further randomised into the saline and RR-11a groups, which ensured comparability of MI injury between the two groups. Of the 60 mice that were subjected to the MI procedure in each group, 28 (46.7%) died in the MI group and 19 (31.7%) in the MI + RR-11a group (*p* = 0.0739); no death occurred in either sham group (*P* = 0.002 among all groups) (Fig. 5A). In mice that died prematurely (non-survivors), the thoracic cavity was opened to identify the cause of death (Fig. 5B). We found that the incidence of cardiac rupture was 31.7% in saline- treated MI mice, which was similar to that reported in previous studies^18,23^. Meanwhile, RR-11a-treated mice showed lower incidence of cardiac rupture (15.0%, *P* = 0.031 compared to saline-treated MI mice; Fig. 5C). Masson’s trichrome staining revealed no difference in the gross morphology of hearts excised from saline or RR-11a-treated sham mice. However, 30 days post-MI, the walls were significantly thicker in the RR-11a-treated compared to those in the saline-treated mice, while the infarct size was similar between the saline and RR-11a-treated mice (Fig. 5D–F). Moreover, the collagen volume fraction was calculated based on picrosirius-red–stained heart sections, which showed that the collagen volume fraction was higher in the infarct and in the infarct border zone in RR-11a-treated mice than that in the saline-treated mice 30 days post-MI (Fig. 5G–I). Accordingly, the amount of collagen fibre in the infarcted area was significantly higher in RR-11a-treated mice than that in saline-treated mice at 30 days post-MI (Fig. 5J). Echocardiography at day 30 post-MI demonstrated that cardiac dimension was significantly reduced (LVEDD: 4.579 ± 0.1696 mm vs. 5.349 ± 0.2391 mm, *P* = 0.0265) and LV function was significantly improved (LVEF: 41.45% ± 4.621% vs. 25.1% ± 3.42%, *P* = 0.0087) in RR-11a-treated MI mice as compared to those in saline-treated MI mice (Fig. 6). These results indicate that legumain inhibition could improve cardiac function and attenuate cardiac remodelling post-MI by decreasing legumain-induced ECM degradation in the MI mouse model.

## Discussion

MI results in ECM modulation and loss of cardiomyocytes. Herein, we show that high legumain concentration is independently associated with increased all-cause mortality in AMI patients, which may be related to cardiac remodelling rather than to cardiomyocyte necrosis. In vitro, legumain treatment resulted in upregulated MMP-2 expression in TGF-β-stimulated fibroblasts, and downregulated ECM components, as represented by fibronectin and collagen content. Inhibition of either legumain or MMP-2 facilitated the recovery of fibronectin and collagen content. In vivo, the legumain inhibitor, RR-11a, markedly improved cardiac function and attenuated cardiac remodelling after MI, while increasing ECM content. Therefore, our data suggest that legumain activation facilitates ECM degradation—at least in part—through an MMP-2 dependent pathway post-AMI. To our knowledge, this is the first report to describe the association between legumain and cardiac remodelling while elucidating the related mechanism in an MI mouse model.

In addition to cardiomyocyte necrosis, ischaemic insult can cause interstitial cell necrosis and excessive ECM degradation, which is strongly associated with cardiac contractility impairment and structure remodelling, or even cardiac rupture post-MI^10^. Growing evidence suggests that ECM changes may play a major role in cardiac remodelling post-MI as well as the extent of myocardial necrosis post-MI^8^. Although myocardial necrosis and heart failure can be estimated based on enzymatic release and brain natriuretic protein levels, respectively, ECM degradation post-MI cannot be readily assessed in clinical settings. Impairment of ECM structure reportedly results in enhanced release and activation of various proteases, which can catalyse the hydrolysis of fibronectin and collagens, hence these proteases may serve as biomarkers of ECM adaptation^3–5^. Here, we demonstrate the following: correlation between PLG and LVEDD; LAD (in a clinical study); degradation of fibronectin and collagen by legumain in vitro; therapeutic effects of legumain inhibitor, RR-11a, for improving cardiac function and attenuating cardiac remodelling post-MI in mice. Cumulatively, these results suggest that plasma legumain may reflect the extent of ECM degradation and that legumain might be a potential therapeutic target in AMI patients.

Circulating legumain level was measured before coronary artery angiography in AMI patients. Higher legumain concentration was associated with increased risk of all-cause mortality during a median follow-up of 39.2 months. Notably, higher legumain concentration remained an independent risk factor for all-cause mortality after adjusting for the potential confounders in multivariate Cox regression. Interestingly, the legumain value corresponded to cardiac chamber remodelling. In addition, the protective effects of anti-legumain therapy were also demonstrated in vivo in the left ventricle chamber and on ejection fraction in MI mice, suggesting that legumain may have a deleterious effect on AMI patients via aggravating cardiac remodelling. We also monitored the concentration of RR-11a after daily intraperitoneal injection and assessed legumain activity and expression post RR-11a treatment and found that the concentration of RR-11a peaked 1.5 h after injection with a plasma half-life of ~5 h. Further, the activity, not the expression of legumain, was significantly suppressed by RR-11a in vivo and in vitro. Hence, the observed beneficial effects of RR-11a in MI mice are primarily related to the suppression of legumain activity. Paradoxically, during the course of the present study, Lunde et al. reported a protective effect of legumain with respect to all-cause mortality; this was attributed to the anti-inflammatory effect of legumain very early after AMI^38^. The reasons for this discrepancy remain unclear; however, the differences in clinical settings may be a contributing factor. For instance, the patients enroled in the current study exhibited higher prevalence of comorbidities, including diabetes mellitus and hypertension, and higher levels of NT-proBNP; however, the patients exhibited lower levels of CRP and LDL (Supplementary Table 3). Furthermore, risk factors other than PLG, such as LVEDD, LAD, BMI, LVEF, and LDL concentration were included as cofounders in the multivariate Cox regression in our study, which demonstrated a role for plasma legumain as an independent predictor for all-cause mortality during a median follow-up of 39.2 months. Future prospective studies involving larger patient cohorts might be helpful to validate the predictive role of plasma legumain and the therapeutic effect of targeting legumain in AMI patients.

The association between legumain and inflammation has been indicated elsewhere^38,39^. In this study, the predictive performance of legumain was better than that of the prototypical inflammatory biomarker CRP—and similar to that of LVEDD and LVEF—with respect to mortality. This observation—in combination with the strong correlation between legumain and NT-proBNP, LVEDD or LAD—suggests that legumain should not be regarded exclusively as an inflammatory factor, but also as a potential biomarker that reflects the degree of cardiac remodelling.

Physiologically, legumain is expressed at low levels in the healthy heart^40^. In the present study, highly increased expression of legumain was observed post-MI, and the elevated legumain levels were primarily observed in the macrophages (Fig. 2E). Legumain expression is increased in macrophages located in tumour tissues, and this phenomenon is associated with ECM degradation and tissue invasion/metastasis^41^. Subsequent studies revealed that legumain is also upregulated in atherosclerotic plaques, and this phenomenon is associated with enhanced plaque instability and rupture^20^. Accordingly, legumain deficient mice exhibited significantly enhanced renal interstitial fibrosis after unilateral ureteral obstruction^19^. Moreover, treatment with legumain inhibitor aggravated fibrotic lesions in a murine model of renal fibrosis^25^. Interestingly, legumain undergoes autoproteolytic maturation in acidic environments for catalytic activation, after which it digests its natural inhibitor, cystatin C, thereby further enhancing its own activation level. In agreement with these findings, we demonstrated that higher activity and expression of legumain were associated with lower fibronectin and collagen content both in vitro and in vivo, and that this was accompanied by thinner chamber walls, dilated cardiac chambers, and higher rate of cardiac rupture post-MI in mice. These results collectively suggest that legumain is involved in cardiac ECM remodelling post-MI.

Metalloproteinases (MMPs)—a family of proteases—are associated with physiological and pathological ECM degradation as they catalyse the irreversible hydrolysis of amide bonds of the ECM^10^. Among the various MMPs, MMP-2 and MMP-15 are the MMP family members that are highly expressed in the myocardium, while MMP-9 is moderately expressed in the heart^42^. However, MMP-2-specific inhibitors or targeted deletion of MMP-2 completely prevent cardiac rupture post-MI in mice^18^, while cardiac rupture was only partially prevented in *MMP-9* knockout mice^43^, suggesting that MMP-2 plays a more deleterious pathological role. Herein, we observed MMP-2 activity to be markedly suppressed in RR-11a-treated fibroblasts and cardiac tissue post-MI. Notably, both RR-11a and MMP-2 inhibitors had similar effects with respect to reversing the degradation of collagen and fibronectin induced by reconstructed legumain in vitro. Hence, legumain-mediated ECM degradation is dependent on the MMP-2 pathway. This result is also in accordance with a former study, that reported that legumain is required for pro-MMP2 maturation and activation through cleavage of the asparaginyl bond in Asn^109^-Tyr^110^ ^17^.

It is also possible that the legumain-mediated degradation is related to non-lysosomal proteolysis, such as the UPS which is present in all eukaryotic cells and is believed to be responsible for non-lysosomal proteolysis^44^ and thus could modulate the remodelling of ECM after MI^45,46^. We thus checked the expression of E3 ubiquitin ligase—a key enzyme in ubiquitination that promotes protein ubiquitination and degradation in the cardiac tissue post-MI—and found that E3 expression was increased in the cardiac tissue post-MI; however, E3 expression was not affected by RR-11a treatment (Supplementary Fig. 8A–B). Furthermore, we examined whether E3 plays a role in legumain-mediated ECM degradation and observed that E3 inhibitor did not impact the level of degradation in vitro (Supplementary Fig. 8C–E). Thus, the ubiquitin pathway does not play a major role in legumain-mediated ECM degradation. However, the detailed association between the UPS and legumain post-MI requires further investigation.

There are several limitations in this study. First, the number of AMI patients was limited, future studies with larger patient cohorts would serve to validate the precise cut-off value to identify patients at high risk of all-cause mortality. Second, we cannot overlook the fact that there might be additional mechanisms associated with legumain-mediated degradation post-MI owing to the pleiotropic nature of legumain, and future studies are warranted to explore the association between legumain and ubiquitination-associated ECM degradation post-MI. Third, the precise regulatory mechanism associated with legumain and BNP in the context of MI requires further investigation.

## Conclusions

In conclusion, our clinical observation as well as our in vivo and in vitro results collectively indicate that legumain is not only a serological biomarker for inflammation and cardiac remodelling, but also a pathogenic element responsible for the poor prognosis of AMI patients. Moreover, its detrimental effects are associated with excessive ECM degradation mediated in part via MMP-2 pathway activation (Supplementary Fig. 10).

## Supplementary information

Supplemental Table

Supplemental Figure 1

Supplemental Figure 2

Supplemental Figure 3

Supplemental Figure 4

Supplemental Figure 5

Supplemental Figure 6

Supplemental Figure 7

Supplemental Figure 8

Supplemental Figure 9

Supplemental Figure 10

Supplementary Figure Legends
