# Supplementary material for: Legumain is a predictor of all-cause mortality and potential therapeutic target in acute myocardial infarction
Source: Cell Death Dis. 2020 Nov 26;11(11):1014. doi: 10.1038/s41419-020-03211-4 (PMC7691341; doi:10.1038/s41419-020-03211-4)
Supplement: Supplementary file 1 — Supplemental Table [file 41419_2020_3211_MOESM1_ESM.docx]

**Supplemental table 1. Plasma legumain concentration in AMI and health control**

| group | Control group | AMI group | P-value |
| --- | --- | --- | --- |
| N | 323 | 212 |  |
| Age (years) | 62.7 ± 13.0 | 60.9 ± 12.7 | 0.117 |
| Male sex | 252 (78.0%) | 166 (78.3%) | 0.938 |
| PLG concentration (μg/L) | 4.4 (3.2-6.1) | 5.9 (4.2-9.3) | <0.001 |
| PLG subgroup |  |  | <0.001 |
| ≤10 μg/L | 311 (96.3%) | 168 (79.2%) |  |
| >10 μg/L | 12 (3.7%) | 44 (20.8%) |  |

Data are described as mean (SD) or median (Q1-Q3)

AMI: acute myocardial infarction; PLG: plasma legumain concentration

**Supplemental Table 2 Comparison of AUC and prediction performance by Harrell’s C index with relative 95% confidence interval (CI)**

| **Comparison Set** | **AUC** | **P Value** | **Harrell’s C index (95%CI interval)** |
| --- | --- | --- | --- |
| CRP *vs* PLG |  |  |  |
| CRP | 0.5109 | 0.0275 | 0.618 (0.5006 -0.7353) |
| PLG | 0.71489 | 0.0145 | 0.6166 (0.5041-0.7291) |
| lnNT-proBNP *vs* PLG |  |  |  |
| lnNT-ProBNP | 0.75816 | **<** 0.0001 | 0.7861 (0.7165- 0.8558) |
| PLG | 0.65293 | 0.0111 | 0.6059 (0.5061- 0.7057) |
| LVEDD *vs* PLG |  |  |  |
| LVEDD | 0.69326 | 0.0260 | 0.6178 (0.51- 0.7256) |
| PLG | 0.66395 | 0.0172 | 0.598 (0.4896- 0.7065) |
| LVEF *vs* PLG |  |  |  |
| LVEF | 0.60334 | 0.0006 | 0.6387 (0.5243- 0.753) |
| PLG | 0.66288 | 0.0187 | 0.5965 (0.4878- 0.7051) |

CRP: C reactive protein (mg/L); PLG: plasma legumain concentration (μg/L); NT-proBNP: N-terminal pro-brain natriuretic peptide (pg/mL); LVEDD: left ventricular end diastolic diameter (mm); LVEF: left ventricular ejection fraction (%)

**Supplemental Table 3. Comparison of baseline clinical characteristic between Lunde’s^3^ and present research**

| Characteristic | Lunde’s research | Present research |
| --- | --- | --- |
| Diabetes mellitus | 17 (6%) | 50 (23.7%) |
| Hypertension | 73 (27%) | 119 (56.1%) |
| Total Cholesterol (mmoL/L) | 5.2 ± 1.1 | 4.1 ± 1.0 |
| NT-proBNP (pg/mL) | 76.3 (42.4-186.4) | 955.3 (417.5-2243.5) |
| CRP (mg/L) | 18 (7-45) | 8.6 (3.8-35.4) |

Continuous normally distributed variables reported as mean±SD, continuous non-normally distributed variables reported as median (IQR). Categorical variables reported as n (%).NT-proBNP: N-terminal pro-brain natriuretic peptide (pg/mL); CRP: C reactive protein (mg/L).
