# Supplementary figures and images for: Legumain is a predictor of all-cause mortality and potential therapeutic target in acute myocardial infarction
Source: Cell Death Dis. 2020 Nov 26;11(11):1014. doi: 10.1038/s41419-020-03211-4 (PMC7691341; doi:10.1038/s41419-020-03211-4)

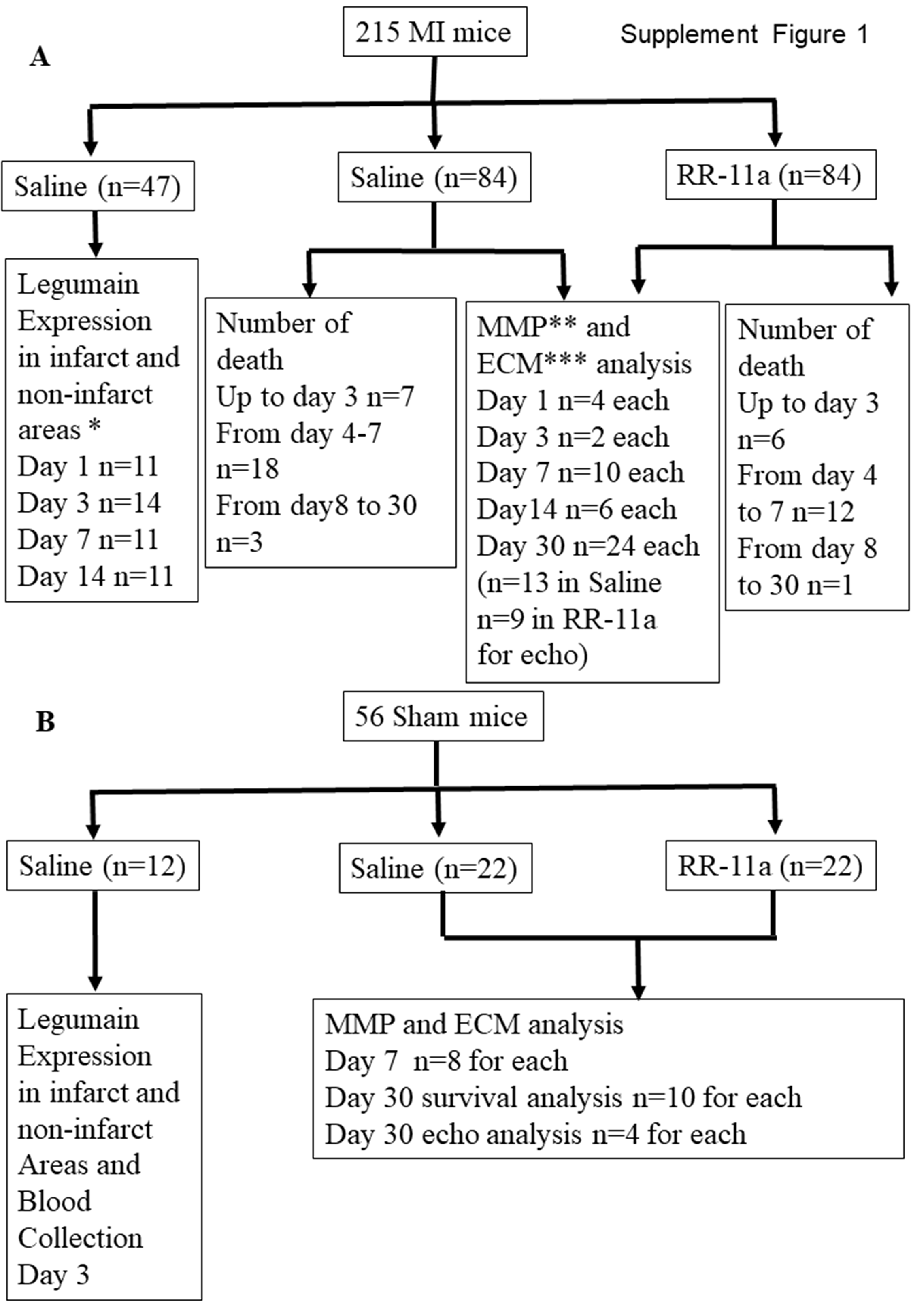

Supplement: Supplementary file 2 — Supplemental Figure 1 [file 41419_2020_3211_MOESM2_ESM.tif]

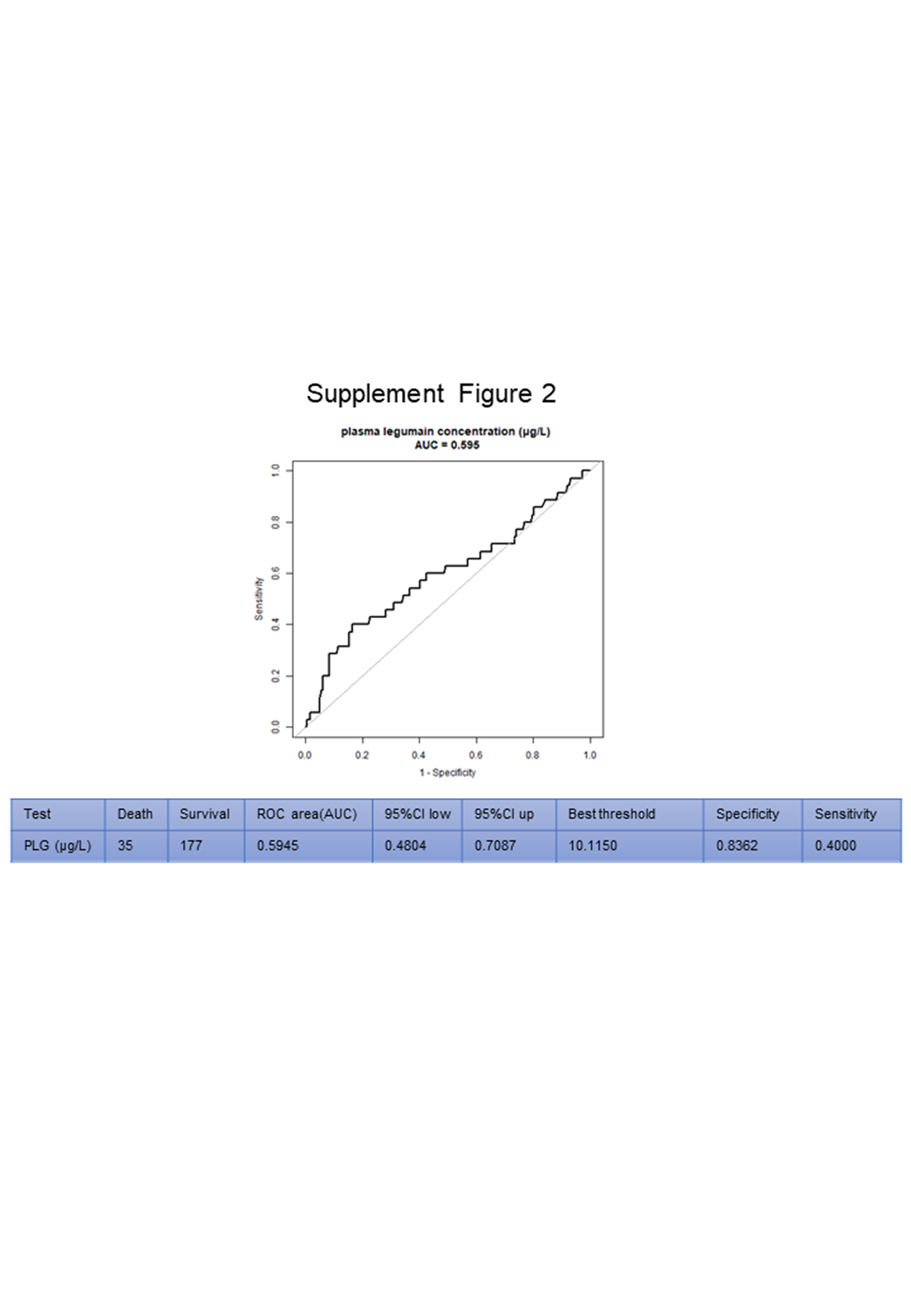

Supplement: Supplementary file 3 — Supplemental Figure 2 [file 41419_2020_3211_MOESM3_ESM.tif]

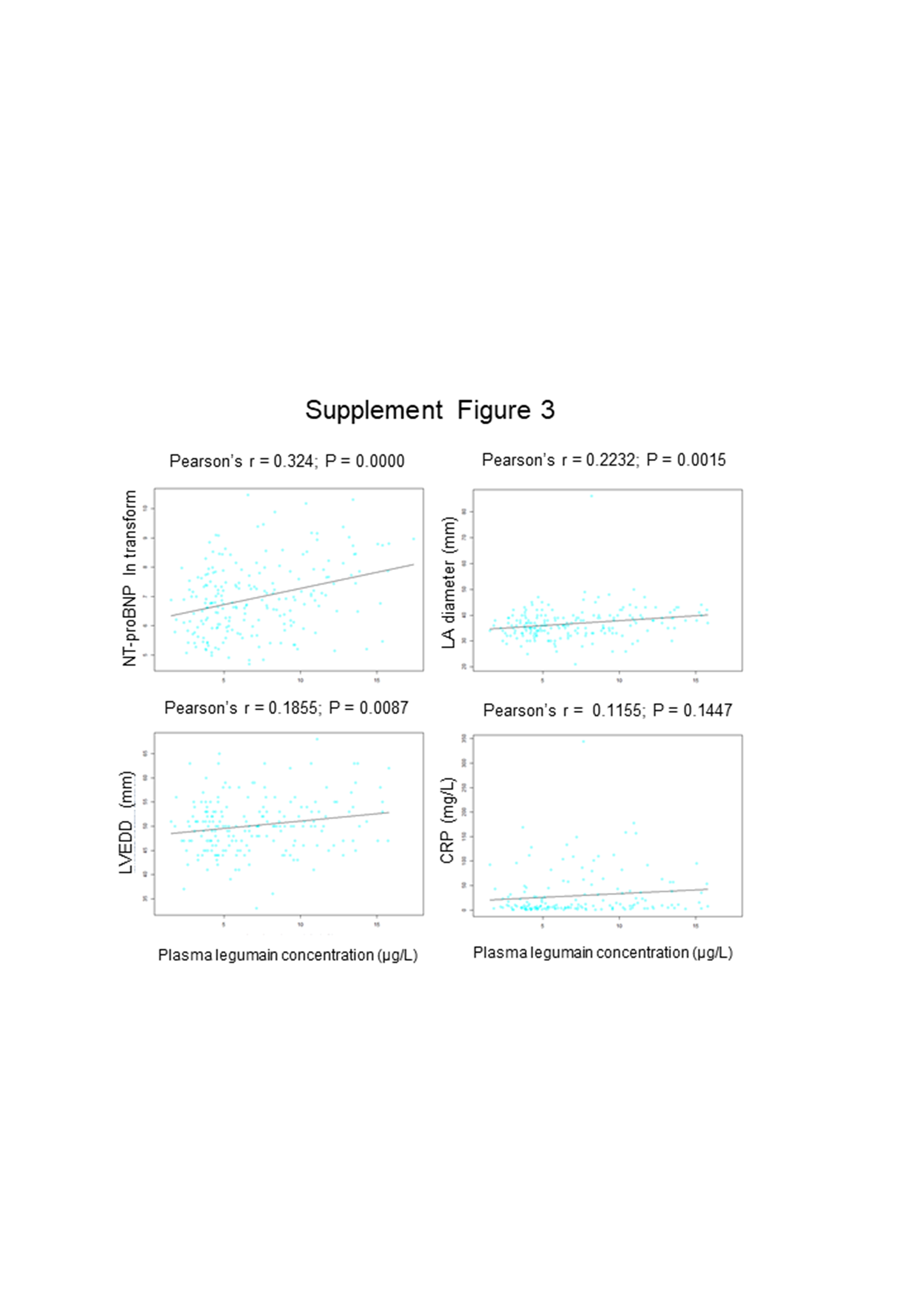

Supplement: Supplementary file 4 — Supplemental Figure 3 [file 41419_2020_3211_MOESM4_ESM.tif]

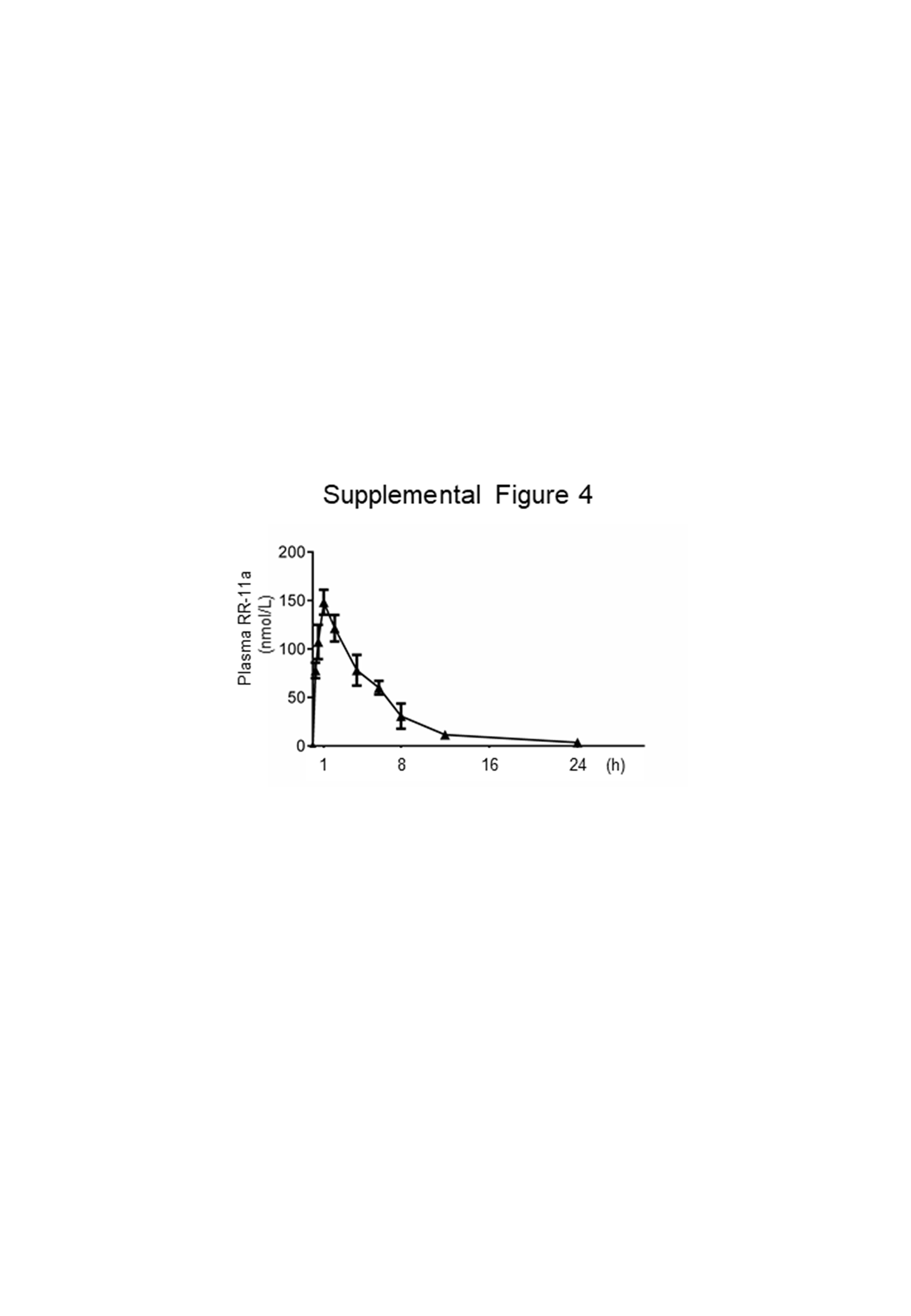

Supplement: Supplementary file 5 — Supplemental Figure 4 [file 41419_2020_3211_MOESM5_ESM.tif]

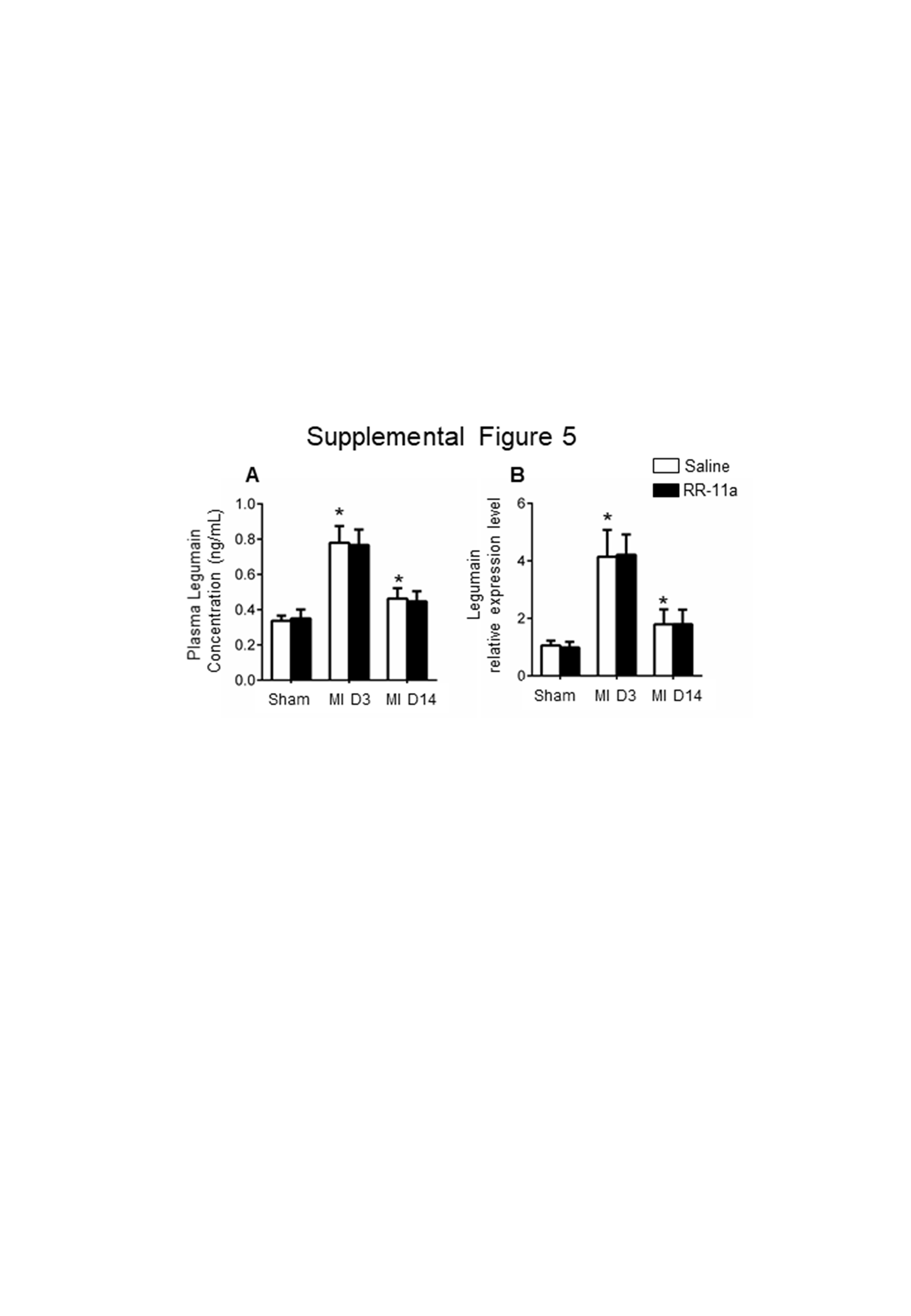

Supplement: Supplementary file 6 — Supplemental Figure 5 [file 41419_2020_3211_MOESM6_ESM.tif]

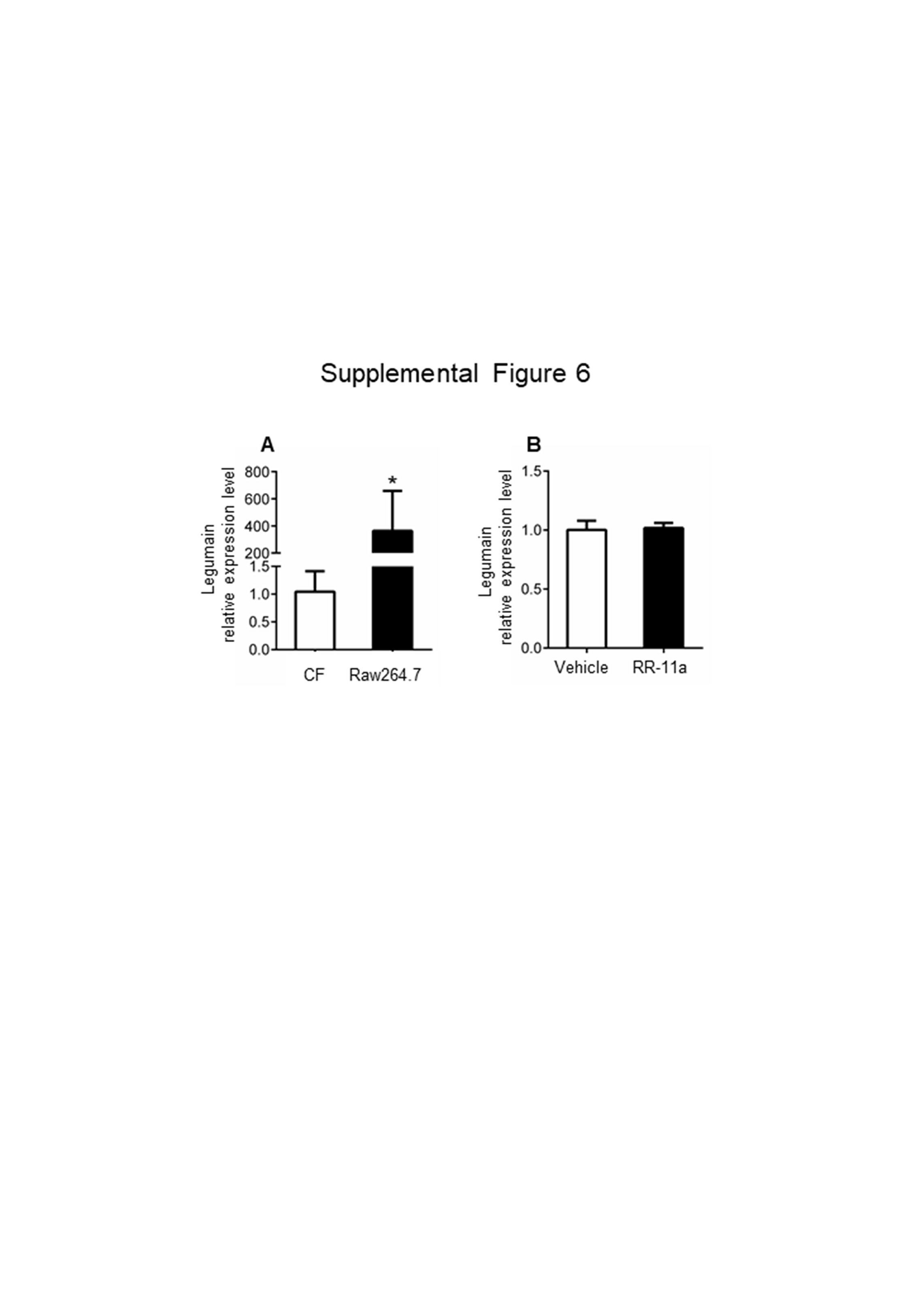

Supplement: Supplementary file 7 — Supplemental Figure 6 [file 41419_2020_3211_MOESM7_ESM.tif]

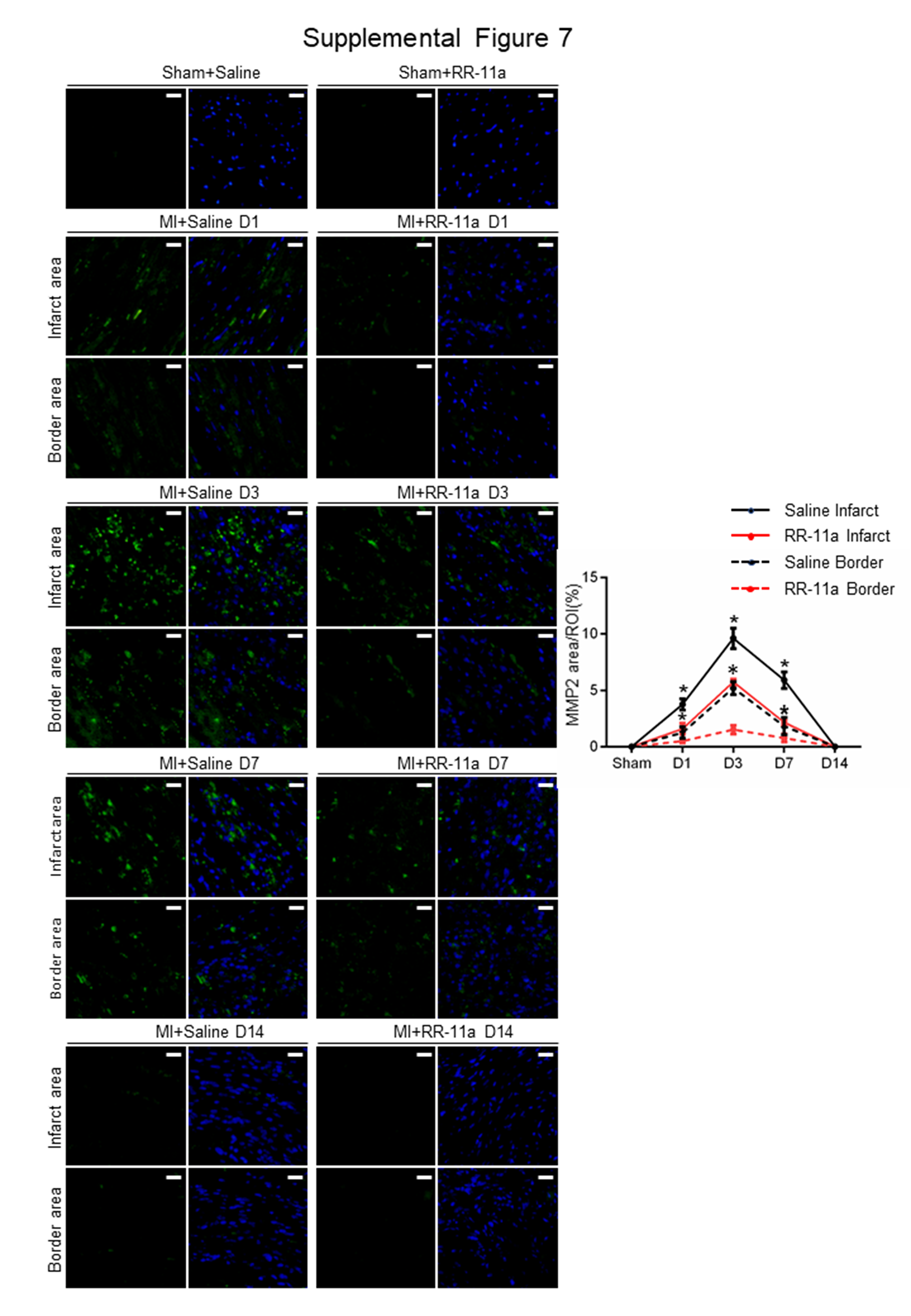

Supplement: Supplementary file 8 — Supplemental Figure 7 [file 41419_2020_3211_MOESM8_ESM.tif]

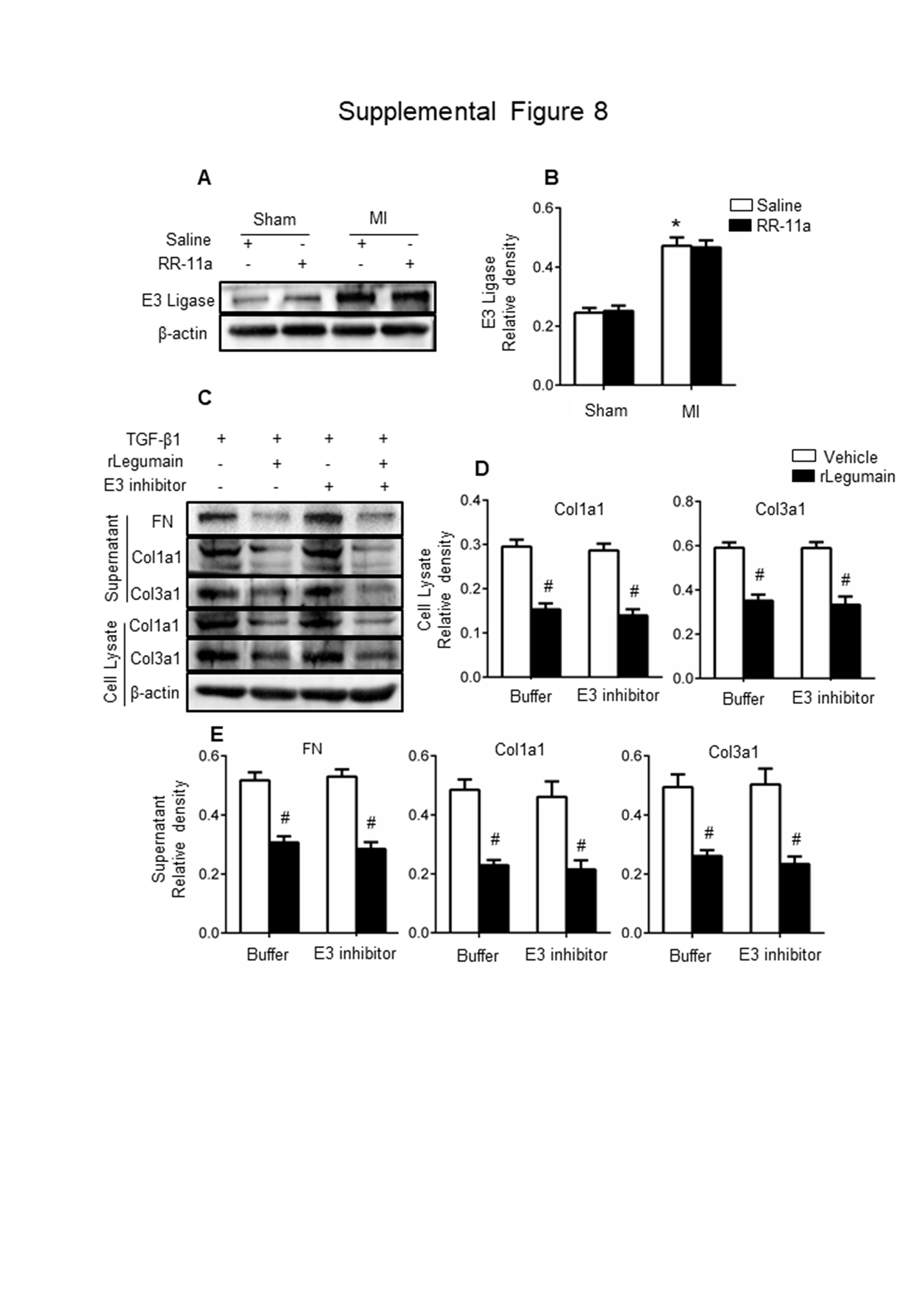

Supplement: Supplementary file 9 — Supplemental Figure 8 [file 41419_2020_3211_MOESM9_ESM.tif]

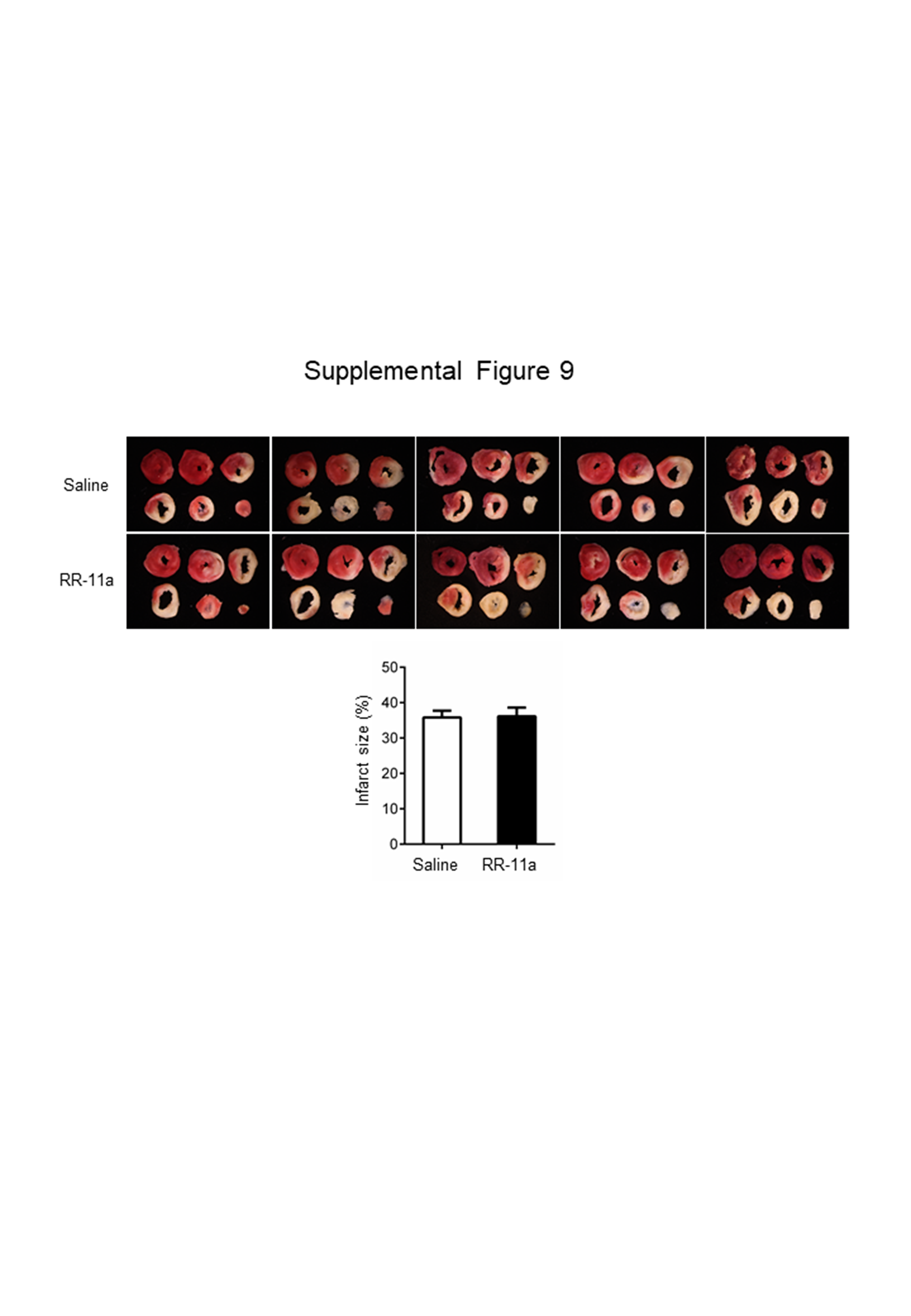

Supplement: Supplementary file 10 — Supplemental Figure 9 [file 41419_2020_3211_MOESM10_ESM.tif]

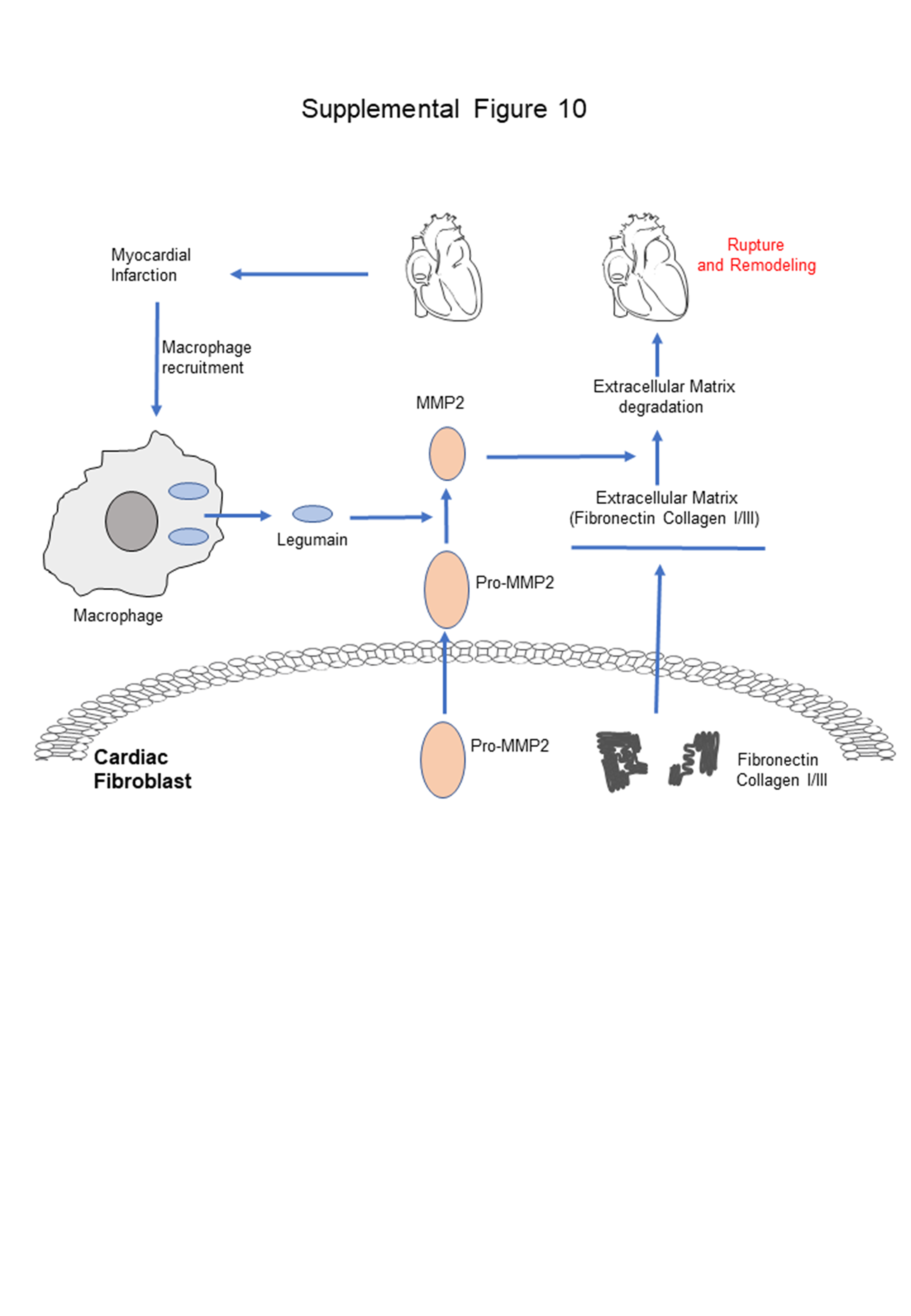

Supplement: Supplementary file 11 — Supplemental Figure 10 [file 41419_2020_3211_MOESM11_ESM.tif]
