# Supplementary material for: Legumain is a predictor of all-cause mortality and potential therapeutic target in acute myocardial infarction
Source: Cell Death Dis. 2020 Nov 26;11(11):1014. doi: 10.1038/s41419-020-03211-4 (PMC7691341; doi:10.1038/s41419-020-03211-4)
Supplement: Supplementary file 12 — Supplementary Figure Legends [file 41419_2020_3211_MOESM12_ESM.docx]

**Supplemental Figure Legends**

**Supplemental Figure 1. Flow chart of the animal study.** *Blood collection was performed at identical time points; **MMP analysis means a gelatin zymogram for MMP-2 activity in cardiac tissues and immunofluorescence staining of MMP-2 at the different time points; ***ECM analysis including α-SMA, collagen I, and collagen III expression at both the mRNA level by RT-qPCR and the protein level by immunofluorescence staining and western blot analysis. Mice surviving at the end of 30 days were randomly selected for echocardiography analysis and then all mice were sacrificed for HE staining, Masson’s trichrome staining and picrosirius red staining and MMP and ECM analysis.

**Supplemental Figure 2. ROC curve of plasma legumain on all-cause mortality.**

**Supplement Figure 3. Correlation of plasma legumain levels with NT-proBNP (on a natural logarithmic scale), LA diameter (mm), LVEDD (mm) and CRP levels.**

**Supplemental Figure 4. The concentration of plasma RR-11a at different time points with the dose of 20mg/Kg RR-11a treatment.**

**Supplemental Figure 5. The expression of legumain in plasma and cardiac tissues after RR-11a treatment. A,** The serum legumain level in mice with or without RR-11a treatment post MI; * p < 0.05 vs sham operation; n = 4. **B,** Quantitative analysis of legumain mRNA levels in mice with or without RR-11a treatment post MI; * p < 0.05 vs sham operation; n = 4–6.

**Supplemental Figure** 6. **The expression level of legumain in cardiac fibroblast.** A, The expression level of legumain in cardiac fibroblast and RAW246.7 without any treatment; * p < 0.05 vs cardiac fibroblast, n = 3; B. The expression level of legumain in cardiac fibroblast with or without RR-11a; * p < 0.05 vs Vehicle treated cardiac fibroblast, n = 3.

**Supplemental Figure 7. Blocking of legumain with RR-11a inhibits matrix metalloproteinase 2 (MMP-2) expression after MI.** Representative images of immunofluorescence staining and quantification analysis of MMP2 levels in cardiac tissues at different time points post-MI; * p < 0.05 vs. saline-treated or RR-11a-treated mice after sham operation; n = 4. Scale bar, 20 μm.

**Supplemental Figure 8. E3 ubiquitin-protein ligase does not play a fundamental role in legumain-mediated ECM degradation.** A-B, Western blot and quantification analysis of E3 ubiquitin-protein ligase level in cardiac tissue at 3 days post-MI, n = 3. * p < 0.05 vs. Sham mice. C-E, Western blot and quantification analysis of fibronectin, collagen I, and collagen III from cell lysates (D) and supernatant (E) in (C), n = 3. #p < 0.05 vs. Vehicle with or without E3 inhibitor treatment.

**Supplemental Figure 9.** LV infarct sizes in mice after MI。Representative images of TTC staining and quantification analysis of infarcted volume 24 hours after left coronary artery ligation. There was no significant difference between Saline and RR-11a group, p=0.8139. (n=5 in each group)

**Supplemental Figure 10. Graph of the role and mechanism of legumain in cardiac rupture and remodelling following myocardial infarction.**
